# Immunoexpression Patterns of Megalin, Cubilin, Caveolin-1, Gipc1 and Dab2IP in the Embryonic and Postnatal Development of the Kidneys in *Yotari* (*Dab1*^−/−^) Mice

**DOI:** 10.3390/biomedicines12071542

**Published:** 2024-07-11

**Authors:** Sani Žužul, Nela Kelam, Anita Racetin, Petra Kovačević, Suzana Konjevoda, Natalija Filipović, Nikola Pavlović, Katarina Vukojević

**Affiliations:** 1 Clinic for Surgery, Clinical Hospital Firule, 21 000 Split, Croatia; sanipenovic@gmail.com; 2 Department of Anatomy, Histology and Embryology, School of Medicine, University of Split, 21 000 Split, Croatia; nela.kelam@mefst.hr (N.K.); amuic@mefst.hr (A.R.); natalija.filipovic@mefst.hr (N.F.); nikolapavlovic326@gmail.com (N.P.); 3 Center for Translational Research in Biomedicine, School of Medicine, University of Split, 21 000 Split, Croatia; 4 Department of Ophthalmology, University Hospital Center of Zagreb, 10 000 Zagreb, Croatia; petra.kovacevic1@kbc-zagreb.hr; 5 Department of Health Studies, University of Zadar, 23 000 Zadar, Croatia; suzanakonjevoda@gmail.com; 6 Department of Ophthalmology, General Hospital Zadar, 23 000 Zadar, Croatia; 7 Department of Anatomy, School of Medicine, University of Mostar, 88 000 Mostar, Bosnia and Herzegovina

**Keywords:** Megalin, Cubilin, Caveolin 1, Gipc1, Dab2IP, CAKUT, *yotari*

## Abstract

Our study examines the immunoexpression patterns of Megalin, Cubilin, Caveolin-1, Gipc1 and Dab2IP in the embryonic development (E) and postnatal (P) mouse kidney, with a focus on differentiating patterns between wild-type (wt) and *yotari*, *Dab1*^−/−^ (*yot*) mice. Immunofluorescence revealed raised immunoexpression of receptors Megalin and Cubilin at the ampulla/collecting ducts and convoluted tubules across all developmental stages, with the most prominent immunoexpression observed in the convoluted tubules and the parietal epithelium of the Bowman’s capsule. Quantitative analysis showed a higher percentage of Megalin and Cubilin in wt compared to *yot* mice at E13.5. Co-expression of Megalin and Cubilin was observed at the apical membrane of convoluted tubules and the parietal layer of the Bowman’s capsule. The staining intensity of Megalin varied across developmental stages, with the strongest reactivity observed at the ampulla and collecting ducts at embryonic day (E) 13.5 in wt mice. In contrast, Caveolin-1 exhibited high immunoexpression in the metanephric mesenchyme, blood vessels, and the border area between the metanephric mesenchyme and renal vesicle, with a decrease in immunoexpression as development progressed. Gipc1 showed diffuse cytoplasmic staining in metanephric mesenchyme, convoluted tubules and collecting ducts, with significant differences in immunoexpression between wild-type and *yot* mice at both investigated embryonic time points. Dab2IP immunofluorescent staining was most prominent in renal vesicle/glomeruli and ampulla/collecting ducts at E13.5, with mild staining intensity observed in the distal convoluted tubules postnatally. Our findings elucidate distinct immunoexpression of patterns and potential parts of these proteins in the development and function of the kidney, highlighting the importance of further investigation into their regulatory mechanisms.

## 1. Introduction

**Scheme 1:**
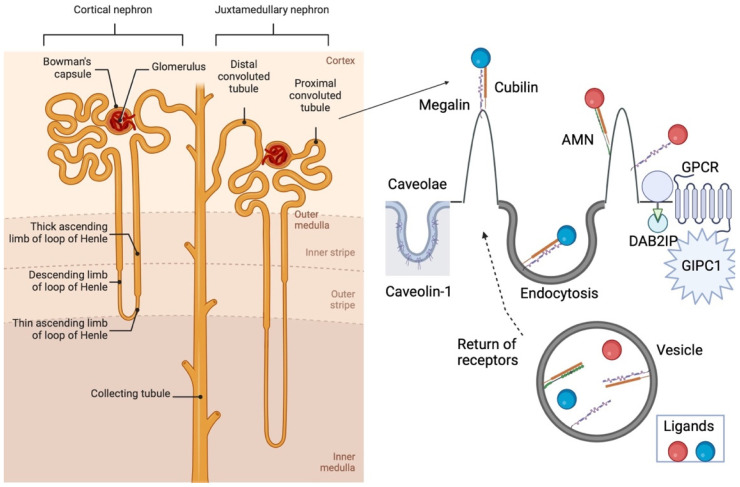
Megalin, Cubilin, Caveolin-1, Gipc1 and Dab2IP are receptors of ligands in kidney structures. In proximal tubular epithelial cells, Megalin, with its cytoplasmic tail, makes a signal for clathrin-mediated endocytosis. Cubilin must join with Megalin or Amnionless (AMN) for successful signaling. After uptake, ligands are relieved in vesicles and transported to the other structures of the cell, while receptors are returned to the apical membrane [20]. Caveolin-1 is the major structure of caveolae in the plasma membrane, which is crucial for cellular metabolism and, consequently, for kidney diseases [27]. Gipc1 interacts with lots of transmembrane receptors (for example, GPCR) for intercellular signaling [28]. Dab2IP is a signaling adaptor that binds with inflammatory cytokines and growth factors [32]. Created with BioRender.com.

The *yotari* (*yot*) mouse is characterized by its mutation of the disabled homolog 1 (Dab1) gene [1], with an abnormality in the central nervous system [1,2,3]. The abnormality is known for its unsteady walk, tremor and premature death when weaning [2]. Dab1, an adapter protein, is important for neuronal layering during embryonic brain development and cell activities in development [4,5,6]. The Dab1 protein can also be found in the peripheral nervous system and some non-neural tissues. For example, it is present in the mouse podocytes and human fetal kidneys [7,8]. We previously discovered that *yotari* mice have renal hypoplasia, a disorder in the spectrum of congenital anomalies of the kidney and urinary tract (CAKUT). Further, *yot* mice have podocyte foot process effacement at the kidney glomeruli that might influence the loss of kidney function [1,9,10]. Therefore, we wanted to investigate different vesicular transport markers to characterize their involvement in kidney function (Scheme 1).

Megalin is a protein member of the low-density lipoprotein (LDL) receptor family [11]. In the kidney, Megalin is revealed in the apical membrane of proximal tubular epithelial cells (PTECs) [12], so it is also known as a receptor of endocytosis [13]. Megalin can be found in different organs, such as the lungs, placenta and brain [14]. Embryonic knockout of Megalin in mice induces a spectrum of abnormalities during the development of the brain and lungs with perinatal death [15]. Megalin interferes with the uptake of essential ligands (like vitamin D) and nephrotoxic substances that can result in kidney injuries [13]. In the apical membrane of PTECs, Megalin interrelates with other proteins, such as Cubilin [16,17], a peripheral glycoprotein lacking transmembrane segments [13].

Cubilin was first described as the receptor of the vitamin B12 complex, but now, many other ligands have been identified [18,19,20]. Cubilin collaborates with Megalin for the endocytic uptake of some ligands [21,22]. The Cubilin gene mutation induces hereditary megaloblastic anemia 1 or Imerslund–Gräsbeck syndrome, characterized by B12 malabsorption and proteinuria [23].

Caveolin-1 is a protein coded by the *CAV1* gene and captures flask-shaped plasma membrane invaginations called caveolae [24]. Caveolae notably increase the cellular surface and are involved in crucial functions of cells, such as transcytosis, endocytosis, signal transduction, lipid homeostasis and chemoprotection [25]. Caveolin-1 has an important role in renal fibrosis [26] and is a potential part of kidney impairment by regulating cellular metabolism [27].

Gipc1 (GAIP-interacting protein, C terminus 1) acts as a PDZ-domain adaptor protein. It helps in the regulation of the cell surface and endocytosis of many transmembrane receptors, signaling and protein complexes [28]. Gipc1 is also responsible for cell division, integrin recycling and the formation of new blood vessels [29]. It is an adaptable molecule responsible for many different cellular and pathophysiological processes. Recently, Gipc1 has been noted for an increased tendency to impact carcinogenesis [28].

Dab2IP (Disabled homolog 2-interacting protein) is a binding protein important for signaling pathways. It has specific cellular functions in modulating signal cascades in cell proliferation, survival, apoptosis and metastasis. Lately, Dab2IP has been found to be significantly downregulated in many types of cancer [30]. It is responsible for immunity, inflammation, chronic stress [31] and cardiovascular disease [32].

This study has the purpose of defining the protein immunoexpression samples of Megalin, Cubilin, Caveolin-1, Gipc1 and Dab2IP in the embryonic (E13.5 and E15.5) and early postnatal (P4 and P14) development of the kidneys in *Dab1*^−/−^ mice compared to wild-type mice. We hypothesize that these proteins are evinced during both the development and postnatal stages in mouse kidneys and that their critical interactions play a significant role in maintaining kidney structure and function.

## 2. Materials and Methods

### 2.1. Ethics

The use of animals in this study was authorized by the Shiga University of Medical Science’s Guidelines for the Care and Use of Laboratory Animals. The research adhered to the principles of the Declaration of Helsinki and received approval from the Ethical Committee of the School of Medicine at the University of Split (class: 003-08/23-03/0015, protocol code no.: 2181-198-03-04-23-0073, approval date: 27 September 2023).

### 2.2. Sample Collection and Generation of Dab1 Null Conventional Mutants

This experiment utilized *yotari* (*Dab1*^−/−^) mice as Dab1 null conventional mutants, developed according to previously described procedures. C57BL/6N mice were bred and group-housed in standard polycarbonate cages, with 3–4 animals per cage, ensuring the representation of each genotype. The mice had unrestricted access to food and tap water in a temperature-controlled room maintained at 23 ± 2 °C. The lighting cycle consisted of 12 h of artificial light and 12 h of darkness. For genotyping, the PCR primers used were *yotari*—GCCCTTCAGCATCACCATGCT and CAGTGAGTACATATTGTGTGAGTTCC, and wild-type of Dab1 locus—GCCCTTCAGCATCACCATGCT and CCTTGTTTCTTTGCTTTAAGGCTGT [33]. Pregnant mice were euthanized on days 13.5 (E13.5) and 15.5 (E15.5) of gestation, and their embryos were collected. Other groups of mice were sacrificed on specific postnatal days, namely 4 and 14. A total of three to four animals were used for each group studied. First, the mice were deeply anesthetized with an intraperitoneal injection of pentobarbital to ensure they were fully unconscious and unresponsive to pain. Afterward, they were transcardially perfused with a phosphate-buffered saline solution (PBS, pH 7.2) to clear the blood from the vascular system and fixated with 4% paraformaldehyde (PFA) in 0.1 M PBS to ensure complete tissue fixation. The kidneys were carefully extracted and treated with 4% PFA in 0.1 M PBS overnight to prepare them for the subsequent conventional histological evaluations, including hematoxylin–eosin and immunofluorescence staining.

### 2.3. Immunofluorescence

The tissue was embedded in paraffin blocks after completing fixation and dehydration using graded ethanol solutions. Sections measuring five microns in thickness were serially cut and subsequently affixed to slides. The tissue integrity of each tenth section was verified by hematoxylin–eosin staining. After deparaffinization in xylol and rehydration in a series of water-ethanol solutions, the mounted tissue samples were steamed in the steam bath (Tefal, Minicompact, Rumilly, Haute-Savoie, France) for 30 min at 95 °C in a 0.01 M citrate buffer (pH 6.0) before being progressively cooled to room temperature. A protein-blocking reagent (ab64226, Abcam, Cambridge, UK) was applied for 20 min following rinsing in 0.1 M PBS to prevent nonspecific staining. Primary antibodies (Table 1) were applied to the sections and left to incubate overnight in a humidity chamber. The next day, after rinsing with PBS, the sections were incubated for an hour with the appropriate secondary antibodies (Table 1). Finally, nuclei were visualized using DAPI (4′,6-diamidino-2-phenylindole) staining after the samples were rinsed in PBS. Samples were dried and cover-slipped (Immuno-Mount, Thermo Shandon, Pittsburgh, PA, USA).

Before conducting the pre-adsorption test, the concentration of each primary antibody in the blocking solution was determined exactly. After adding an appropriate peptide antigen, the resulting mixture was applied to the sections. The results had no evidence of any antibody staining. The absence of primary antibodies in the immunofluorescence protocol did not result in the emergence of false-positive results or nonspecific secondary antibody binding.

### 2.4. Data Acquisition and Analysis

The sections were analyzed using a Nikon DS-Ri2 camera (Nikon Corporation, Tokyo, Japan) in conjunction with an immunofluorescence microscope (BX51, Olympus, Tokyo, Japan). To quantify the immunoexpression of target proteins, constant exposure times were analyzed while non-overlapping visual fields were captured at 40× magnification. A minimum of ten images depicting substructures of the kidney during embryonic development were obtained: metanephric mesenchyme (mm); immature glomeruli (g); renal vesicles (rv); ampullae (A), convoluted tubules (Ct); and collecting ducts (Cd) at embryonic days of E13.5 and E15.5. Additionally, twenty images were obtained of kidney structures during postnatal development, including glomeruli (g), proximal convoluted tubules (pct) and distal convoluted tubules (dct) on postnatally at days P4 and P14. *Lotus tetragonolobus* lectin (LTL) was used as a marker for proximal tubules, *Dolichos biflorus* agglutinin (DBA) as a marker for developing renal collecting ducts and distal tubules in the postnatal kidney cortex, and Aquaporin 2 (AQP2) as a marker for developing collecting duct stalks and distal tubules and collecting tubules in the postnatal kidney cortex as presented in Supplementary Material. Adobe Photoshop version 21.0.2 (Adobe, San Jose, CA, USA) and ImageJ software version 1.530 (National Institutes of Health, Bethesda, MD, USA) were used to analyze each captured image. The number of immunoreactive cells for Megalin, Cubilin, Caveolin, Gipc-1 and Dab2IP was determined, and the resulting percentage of total cells was averaged across all animal groups. Positive results were considered for any degree of cytoplasmic, nuclear or membrane staining with the employed markers. In consideration of variations between observers, the captured microphotographs were independently analyzed by three investigators. Interclass correlation analysis demonstrated interrater agreement with a coefficient greater than 0.8, signifying outstanding agreement [34].

Four degrees of semi-quantitative evaluation were applied to the staining intensities of distinct kidney structures: no reactivity (−); mild reactivity (+); moderate reactivity (++); and strong reactivity (+++) (Table 2 and Table 3). The semi-quantitative evaluation was conducted by an expert histologist who carefully examined the sections and provided the data.

### 2.5. Statistical Analyses

For statistical analyses, GraphPad Prism 8.0.1 was utilized (GraphPad Software, San Diego, CA, USA). The normality of the data distribution was assessed using the Shapiro–Wilk test. Immunoexpression of the target proteins, Megalin, Cubilin, Caveolin-1, Gipc1 and Dab2IP was compared using a two-way ANOVA followed by Tukey’s multiple comparison test to identify significant differences in the percentage of positive cells between E15.5 and E13.5 for mm, g/rv, Ct and A/Cd and P4 and P14 for g, pct and dct. The two parameters used in the ANOVA analysis were the phenotype combined with the observed time point (wild-type mice and *yotari* on E13.5, 15.5 or P4 and P14) and the observed substructures of the kidney (mm, rv/g, A/Cd, Ct, G, PCT and DCT). The expression for the percentage of positive cells was mean + standard deviation (SD). The level of significance was established at *p* < 0.05.

## 3. Results

### 3.1. Megalin and Cubilin Immunoexpression

**Figure 1:**
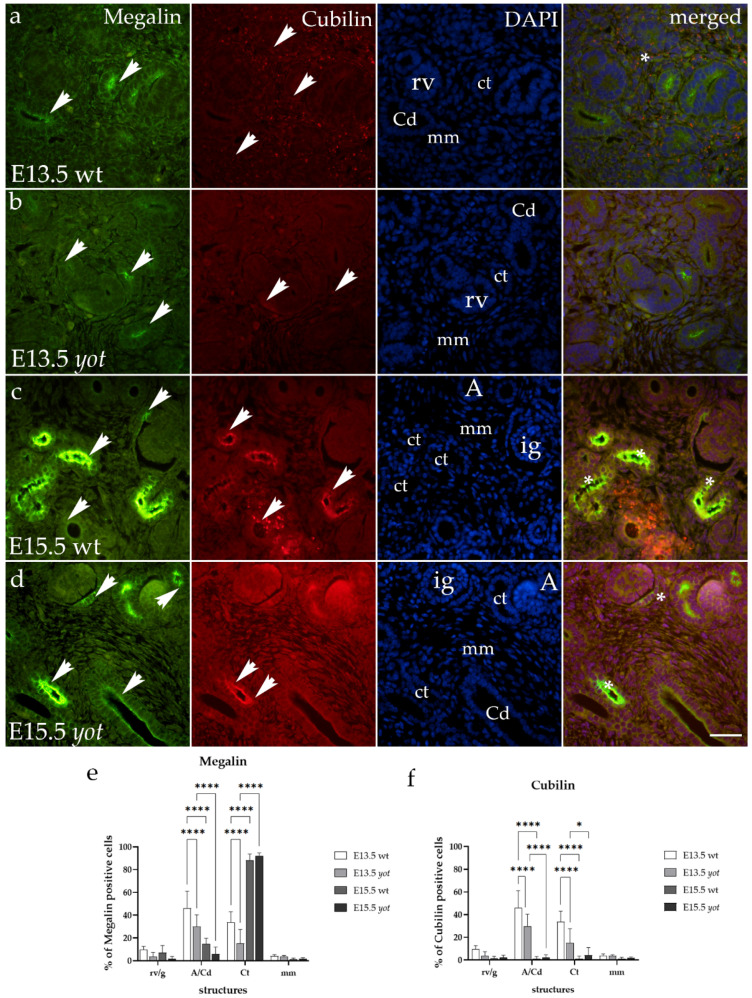
Double immunofluorescence staining of embryonic wild-type and *yotari* mouse kidneys was performed using Megalin (**a**–**d**) and Cubilin (**a**–**d**) markers. Arrows indicate the immunoexpression patterns of Megalin and Cubilin in the metanephric mesenchyme (mm), glomeruli (g), renal vesicles (rv), ampullae (A), convoluted tubules (Ct) and collecting ducts (Cd), as shown on the 4′,6-diamidino-2-phenylindole (DAPI) nuclei staining image. Immunoexpression of Megalin, Cubilin, DAPI staining and the merged images of Megalin, Cubilin and DAPI was observed at embryonic days E13.5 and E15.5 in wild-type (**a**,**c**) and *yotari* (**b**,**d**) kidneys. An asterisk marks the region where co-expression was detected. Images were taken at ×40 magnification, and the scale bar represents 100 μm for all images. The percentages of Megalin (**e**) and Cubilin (**f**) positive cells in the metanephric mesenchyme (mm), renal vesicles (rv) or glomeruli (g), convoluted tubules (Ct) and ampullae (A) or collecting ducts (Cd) of wild-type and *yotari* kidneys at embryonic days 13.5 and 15.5 are shown. Data are presented as the mean ± SD (vertical line) and analyzed using a two-way ANOVA followed by Tukey’s multiple comparison test. Significant differences are indicated by * *p* < 0.05 and **** *p* < 0.0001. Ten substructures were assessed at each time point.

Megalin and Cubilin were highly immunoexpressed in the ampulla/collecting ducts and convoluted tubules in all examined stages (Figure 1a–d and Figures S1 and S3). Megalin and Cubilin immunoexpression was most prominent in the convoluted tubules and the Bowman’s capsule’s parietal layer (Figure 1c,d). The percentage of cells positive for Megalin at E13.5 was higher in wild-type (wt) in ampulla/collecting ducts and convoluted tubules in comparison to *yot* (*p* < 0.0001) (Figure 1e). At E15.5, Megalin immunoexpression was higher in the ampulla/collecting ducts and convoluted tubules of wt than in *yot*, but without statistical significance. The percentage of cells positive for Cubilin at E13.5 was higher in wt in ampulla/collecting ducts and convoluted tubules in comparison to *yot* (*p* < 0.0001) (Figure 1e). At E15.5, the immunoexpression of Cubilin was higher in the ampulla/collecting ducts and convoluted tubules of *yot* than at wt, but it was without statistical significance.

It was observed that the immunoexpression of Megalin and Cubilin decreased as development progressed at the ampulla/collecting ducts, while in convoluted tubules, there was a different pattern with an increase in Megalin and a decrease in Cubilin immunoexpression (Figure 1e). Co-expression of Megalin and Cubilin was observed at the apical membrane of convoluted tubules and the parietal epithelium of the Bowman’s capsule (Figure 1c,d).

The staining intensity of Megalin in wt mice at E13.5 was strongest in the ampulla/collecting duct, while in *yot*, we observed moderate staining intensity in all observed structures. At E15.5, the strongest reactivity was found in convoluted tubules of wt mice and ampulla/collecting ducts in *yot* (Table 2).

**Figure 2:**
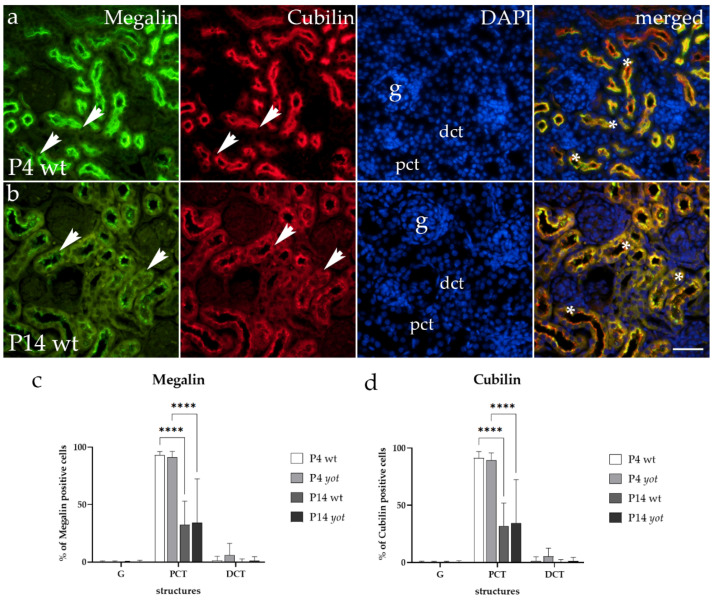
Double immunofluorescence staining of postnatal wild-type and *yotary* mouse kidneys was performed using Megalin (**a**,**b**) and Cubilin (**a**,**b**) markers. Arrows show the immunoexpression patterns of Megalin and Cubilin in glomeruli (g), distal convoluted tubules (dct) and proximal convoluted tubules (pct), indicated on 4′,6-diamidino-2-phenylindole (DAPI) nuclei staining image. Immunoexpression of Megalin and Cubilin staining and merged Megalin, Cubilin and DAPI at 4 days (P4) and 14 days (P14) in wild type. Wild-type and *yotari* mice at postnatal days 4 and 14 of kidney development predominantly matched the localization and intensity of Megalin and Cubilin. Therefore, representative images were taken from wild-type mice. An asterisk marks the region where co-expression was detected. Images were taken at ×40 magnification, and the scale bar represents 100 μm for all images. The percentages of Megalin (**c**) and Cubilin (**d**) positive cells in the glomeruli (G), distal convoluted tubules (DCT) and proximal convoluted tubules (PCT) of postnatal kidneys of wild type and *yotari* animals over time (P4 and P14). Data are presented as the mean ± SD (vertical line) and analyzed using a two-way ANOVA followed by Tukey’s multiple comparison test. Significant differences are indicated by **** *p* < 0.0001. Twenty substructures were assessed at each time point.

In the postnatal developmental stages, Megalin and Cubilin staining was most prominent in the apical membranes of cells of proximal convoluted tubules (Figure 2a,b and Figures S2 and S4). The percentage of Megalin and Cubilin positive cells was highest at P4 in both wt and *yot* mice and decreased statistically in the later developmental stage (P14) (Figure 2c,d). No significant difference was observed between wt and *yot* mice in all observed structures at all developmental stages (Figure 2c,d). Co-expression of Megalin and Cubilin was noticed at proximal convoluted tubules (Figure 2a,b).

Concerning staining intensity, the highest reactivity was noted in proximal convoluted tubules in both phenotypes at all postnatal developmental stages (Table 3).

### 3.2. Caveolin-1 Immunoexpression

**Figure 3:**
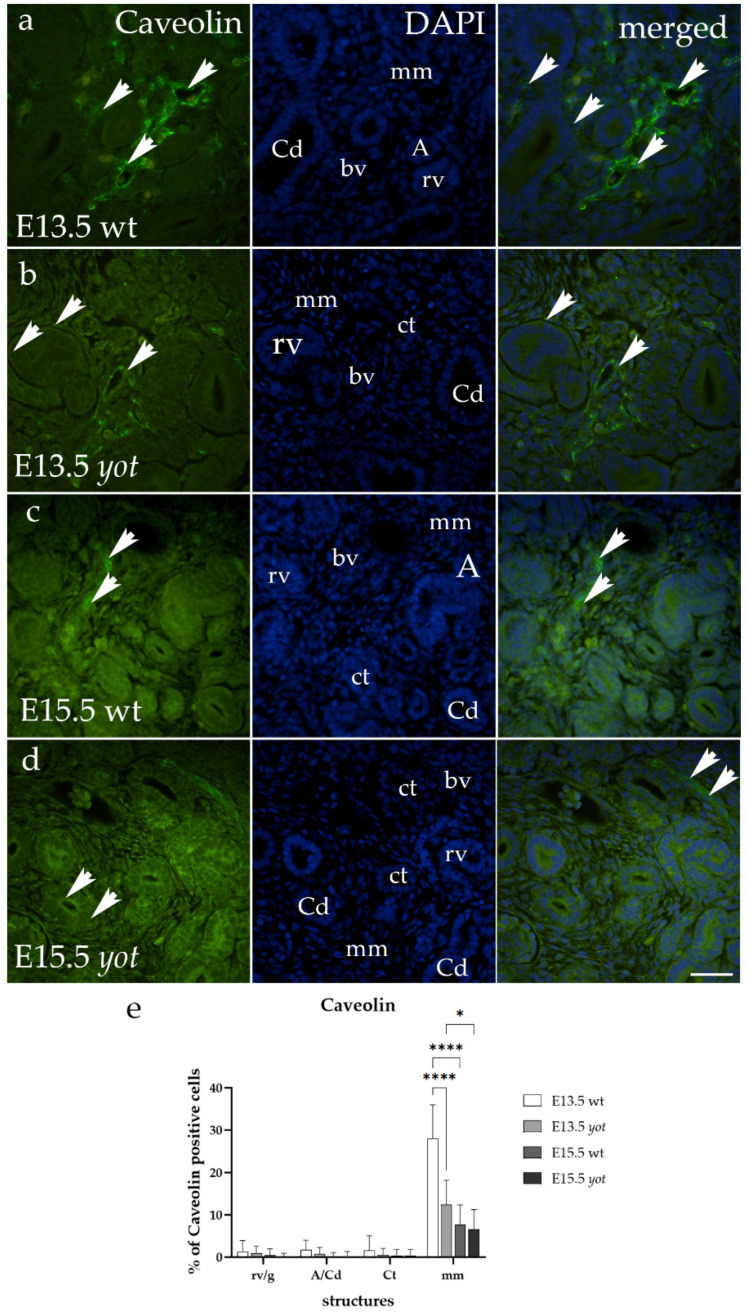
Immunofluorescence staining of embryonic wild-type and *yotari* mouse kidneys was performed using the Caveolin-1 (**a**–**d**) marker. Arrows indicate the immunoexpression patterns of Caveolin-1 in the metanephric mesenchyme (mm), glomeruli (g), renal vesicles (rv), ampullae (A), convoluted tubules (Ct) and collecting ducts (Cd), as shown on the 4′,6-diamidino-2-phenylindole (DAPI) nuclei staining image. Immunoexpression of Caveolin-1, DAPI staining and the merged images of Caveolin-1 and DAPI was observed at embryonic days E13.5 and E15.5 in wild-type (**a**,**c**) and *yotari* (**b**,**d**) kidneys. Images were taken at ×40 magnification, and the scale bar represents 100 μm for all images. The percentages of Caveolin-1 (**e**) positive cells in the metanephric mesenchyme (mm), renal vesicles (rv) or glomeruli (g), convoluted tubules (Ct) and ampullae (A) or collecting ducts (Cd) of wild-type and *yotari* kidneys at embryonic days 13.5 and 15.5 are shown. Data are presented as the mean ± SD (vertical line) and analyzed using a two-way ANOVA followed by Tukey’s multiple comparison test. Significant differences are indicated by * *p* < 0.05 and **** *p* < 0.0001. Ten substructures were assessed at each time point.

High immunoexpression of Caveolin-1 was observed in metanephric mesenchyme, in the blood vessels and border area between the metanephric mesenchyme and renal vesicle in all examined stages (Figure 3a–d and Figure S5). The share of Caveolin-1 positive cells at E13.5 and E15.5 was higher in wt in metanephric mesenchyme in comparison to *yot* (*p* < 0.0001 and *p* < 0.001, respectively) (Figure 3e). The immunoexpression of Caveolin-1 decreased with development progression (Figure 3e). The staining intensity was strongest in the metanephric mesenchyme in both phenotypes at all examined stages (Table 2).

**Figure 4:**
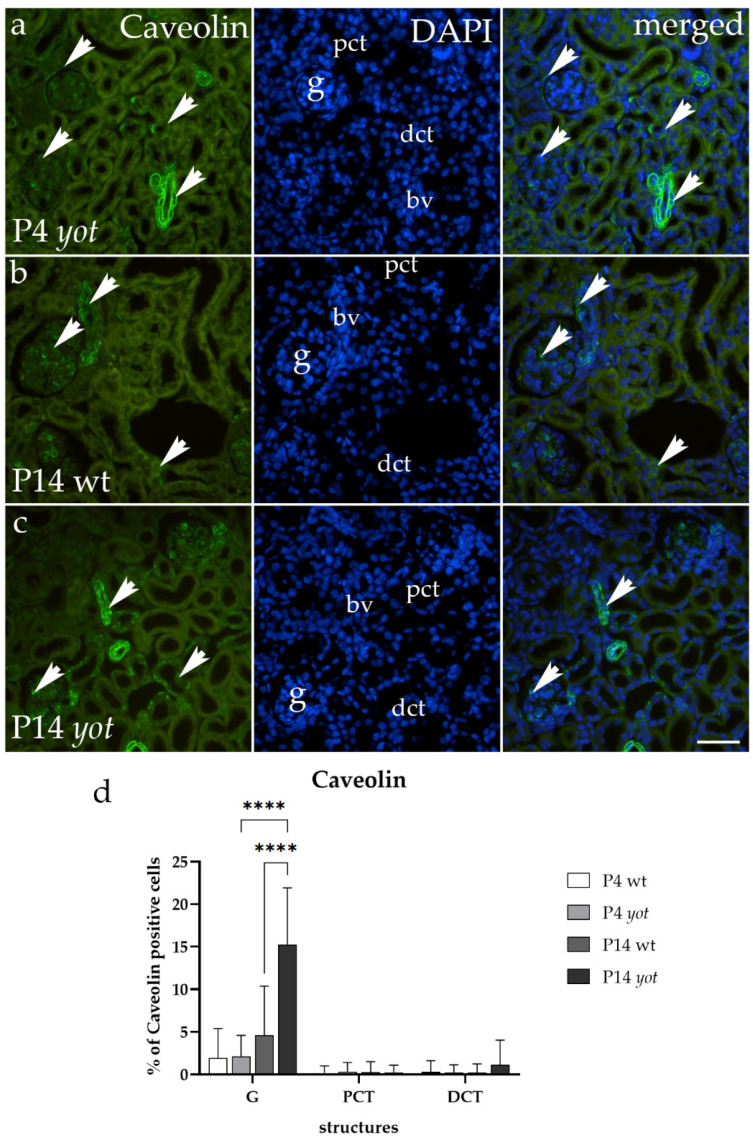
Immunofluorescence staining of postnatal wild-type and *yotari* mouse kidneys was performed using Caveolin-1 (**a**–**c**) markers. Arrows show the immunoexpression patterns of Caveolin-1 in glomeruli (g), distal convoluted tubules (dct) and proximal convoluted tubules (pct), indicated on 4′,6-diamidino-2-phenylindole (DAPI) nuclei staining image. Immunoexpression of Caveolin-1 staining and merged Caveolin-1 and DAPI at 4 days (P4) and 14 days (P14) in wild type (**b**) and *yotari* (**a**,**c**) mice. Wild-type and *yotari* mice at postnatal day 4 of kidney development predominantly matched the localization and intensity of Caveolin-1. Therefore, representative images were taken from wild-type mice. Images were taken at ×40 magnification, and the scale bar represents 100 μm for all images. The percentages of Caveolin-1 positive cells (**d**) positive cells in the glomeruli (G), distal convoluted tubules (DCT) and proximal convoluted tubules (PCT) of postnatal kidneys of wild type and *yotari* animals over time (P4 and P14). Data are presented as the mean ± SD (vertical line) and analyzed using a two-way ANOVA followed by Tukey’s multiple comparison test. Significant differences are indicated by **** *p* < 0.0001. Twenty substructures were assessed at each time point.

In the postnatal developmental stages, Caveolin-1 staining was most prominent in the blood vessels, glomeruli and the parietal layer of the Bowman’s capsule (Figure 4a–c and Figure S6). The share of Caveolin-1 positive cells was highest at P14 in both wt and *yot* mice, and there was a statistically significant difference between P14 wt and *yot* (Figure 4d). There was also a statistically significant difference in *yot* mice compared to wt at P14 (*p* < 0.00001).

The staining intensity of Calveolin-1 was strongest in the glomeruli of both phenotypes at all examined stages, while other structures were negative (Table 2).

### 3.3. Gipc1 Immunoexpression

**Figure 5:**
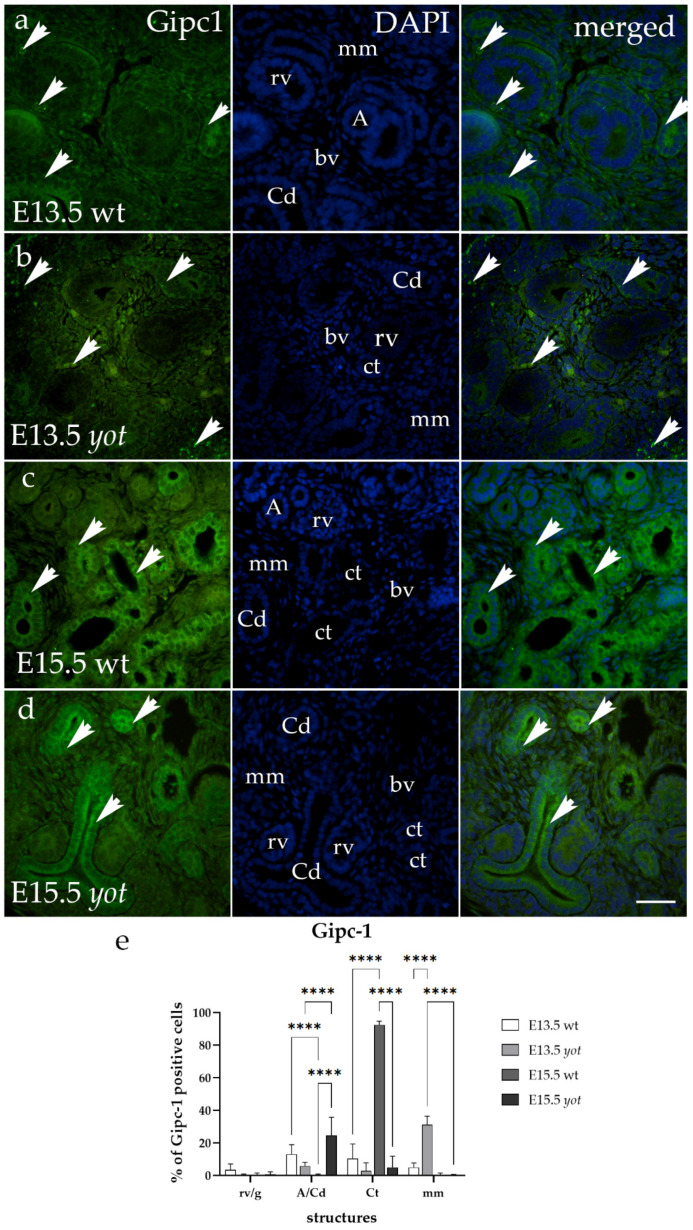
Immunofluorescence staining of embryonic wild-type and *yotari* mouse kidneys was performed using the Gipc1 (**a**–**d**) marker. Arrows indicate the immunoexpression patterns of Gipc1 in the metanephric mesenchyme (mm), glomeruli (g), renal vesicles (rv), ampullae (A), convoluted tubules (Ct) and collecting ducts (Cd), as shown on the 4′,6-diamidino-2-phenylindole (DAPI) nuclei staining image. Immunoexpression of Gipc1, DAPI staining and the merged images of Gipc1 and DAPI was observed at embryonic days E13.5 and E15.5 in wild-type (**a**,**c**) and *yotari* (**b**,**d**) kidneys. Images were taken at ×40 magnification, and the scale bar represents 100 μm for all images. The percentages of Gipc1 (**e**) positive cells in the metanephric mesenchyme (mm), renal vesicles (rv) or glomeruli (g), convoluted tubules (Ct) and ampullae (A) or collecting ducts (Cd) of wild-type and *yotari* kidneys at embryonic days 13.5 and 15.5 are shown. Data are presented as the mean ± SD (vertical line) and analyzed using a two-way ANOVA followed by Tukey’s multiple comparison test. Significant differences are indicated by **** *p* < 0.0001. Ten substructures were assessed at each time point.

Gipc1 was observed as diffuse cytoplasmic staining in metanephric mesenchyme, convoluted tubules and collecting ducts (Figure 5a–d and Figure S7). The part of positive Gipc1 cells at E13.5 was highest in *yot* in metanephric mesenchyme in comparison to wt (*p* < 0.0001) (Figure 5e). At E15.5, the percentage of positive Gipc1 cells was highest in *yot* in ampulla/collecting ducts compared to wt mice (Figure 5e). Additionally, we observed a statistically significant increase in the number of Gipc1 positive cells in convoluted tubules of wt mice compared to *yot* (*p* < 0.0001) (Figure 5e). The staining intensity was strongest in the metanephric mesenchyme in both phenotypes at E13.5 (Table 2). In the later developmental stage, moderate staining reactivity was noticed in convoluted tubules of wt and ampulla/collecting ducts of *yot* mice (Table 2).

**Figure 6:**
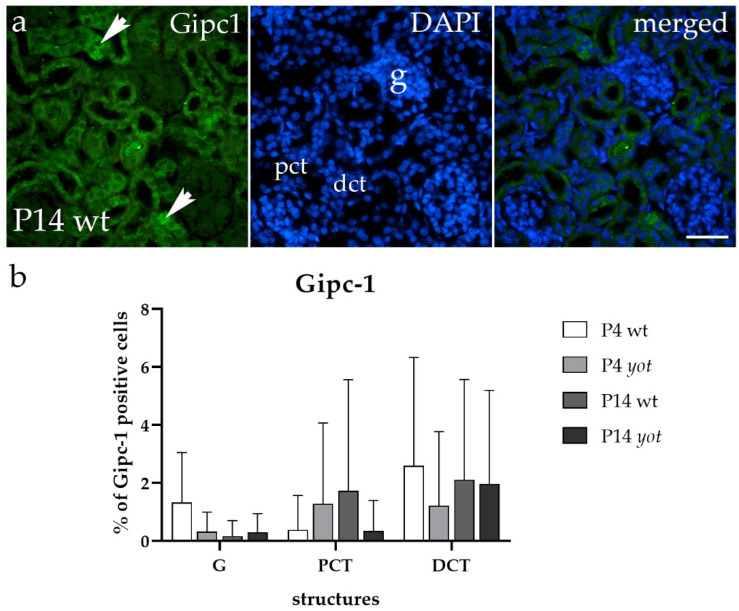
Immunofluorescence staining of postnatal wild-type and *yotari* mouse kidneys was performed using the Gipc1 (**a**) marker. Arrows show the immunoexpression patterns of Gipc1 in glomeruli (g), distal convoluted tubules (dct) and proximal convoluted tubules (pct), indicated on 4′,6-diamidino-2-phenylindole (DAPI) nuclei staining image. Immunoexpression of Gipc1 staining and merged Gipc1 and DAPI at 14 days (P14) in wild-type mice. Wild-type and *yotari* mice at postnatal days 4 and 14 of kidney development predominantly matched the localization and intensity of Caveolin-1. Therefore, representative images were taken from wild-type mice. Images were taken at ×40 magnification, and the scale bar represents 100 μm for all images. The percentages of Gipc1 positive cells (**b**) positive cells in the glomeruli (G), distal convoluted tubules (DCT) and proximal convoluted tubules (PCT) of postnatal kidneys of wild type and *yotari* animals over time (P4 and P14). Data are presented as the mean ± SD (vertical line) and analyzed using a two-way ANOVA followed by Tukey’s multiple comparison test. Twenty substructures were assessed at each time point.

In the postnatal period, Gipc1 was observed as a punctuative signal in the cytoplasm of all observed structures (Figure 6a and Figure S8). There was no statistically significant difference in observed structures between examined phenotypes at all observed time points (Figure 6b). Mild staining intensity of Gipc1 was observed in glomeruli and distal convoluted tubules in wt mice at P4 and P14 (Table 3.). In *yot* mice, the mild staining intensity of Gipc1 was noticed in convoluted tubules (Table 3).

### 3.4. Dab2IP Immunoexpression

**Figure 7:**
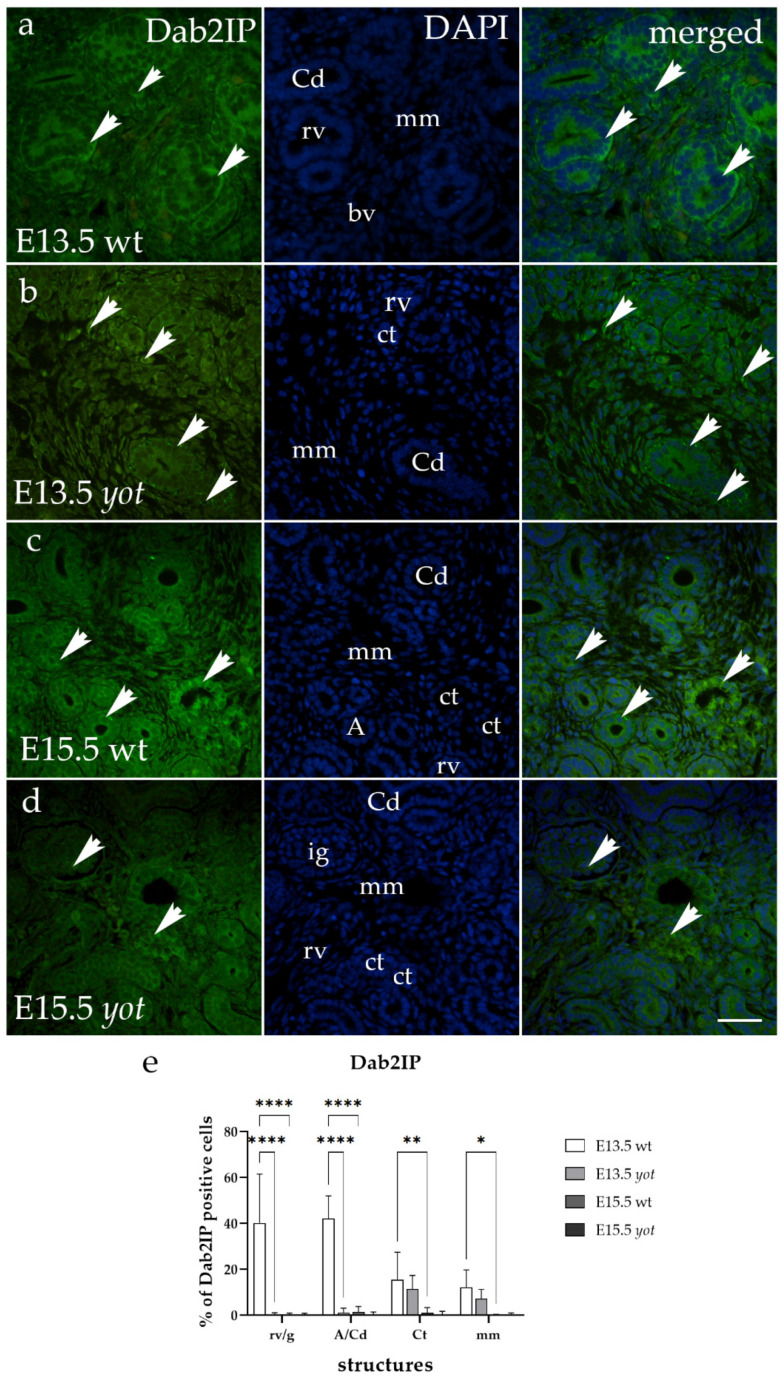
Immunofluorescence staining of embryonic wild-type and *yotari* mouse kidneys was performed using the Dab2IP (**a**–**d**) marker. Arrows indicate the immunoexpression patterns of Dab2IP in the metanephric mesenchyme (mm), glomeruli (g), renal vesicles (rv), ampullae (A), convoluted tubules (Ct) and collecting ducts (Cd), as shown on the 4′,6-diamidino-2-phenylindole (DAPI) nuclei staining image. Immunoexpression of Dab2IP, DAPI staining and the merged images of Gipc1 and DAPI was observed at embryonic days E13.5 and E15.5 in wild-type (**a**,**c**) and *yotari* (**b**,**d**) kidneys. Images were taken at ×40 magnification, and the scale bar represents 100 μm for all images. The percentages of Dab2IP (**e**) positive cells in the metanephric mesenchyme (mm), renal vesicles (rv) or glomeruli (g), convoluted tubules (Ct) and ampullae (A) or collecting ducts (Cd) of wild-type and *yotari* kidneys at embryonic days 13.5 and 15.5 are shown. Data are presented as the mean ± SD (vertical line) and analyzed using a two-way ANOVA followed by Tukey’s multiple comparison test. Significant differences are indicated by * *p* < 0.05, ** *p* < 0.01, **** *p* < 0.0001. Ten substructures were assessed at each time point.

The most prominent Dab2IP staining was observed in renal vesicle/glomeruli and ampulla/collecting ducts in wt mice at E13.5 (Figure 7a,c and Figure S9). In *yot*, staining was noticed in convoluted tubules and metanephric mesenchyme in the same developmental stage (Figure 7b,d). At E15.5, there was no Dab2IP reactivity in any structures or any observed stages (Figure 7e). We observed a statistically significant difference in the percentage of Dab2IP positive cells in renal vesicle/glomeruli and ampulla/collecting ducts of wt mice compared to *yot* at E13.5 (*p* < 0.0001) (Figure 7e). A mild staining intensity was observed in both phenotypes and all structures at E13.5 (Table 2). In the later developmental stage, mild staining reactivity was noticed in convoluted tubules and ampulla/collecting ducts at wt mice, while no reactivity was observed in *yot* mice (Table 2).

**Figure 8:**
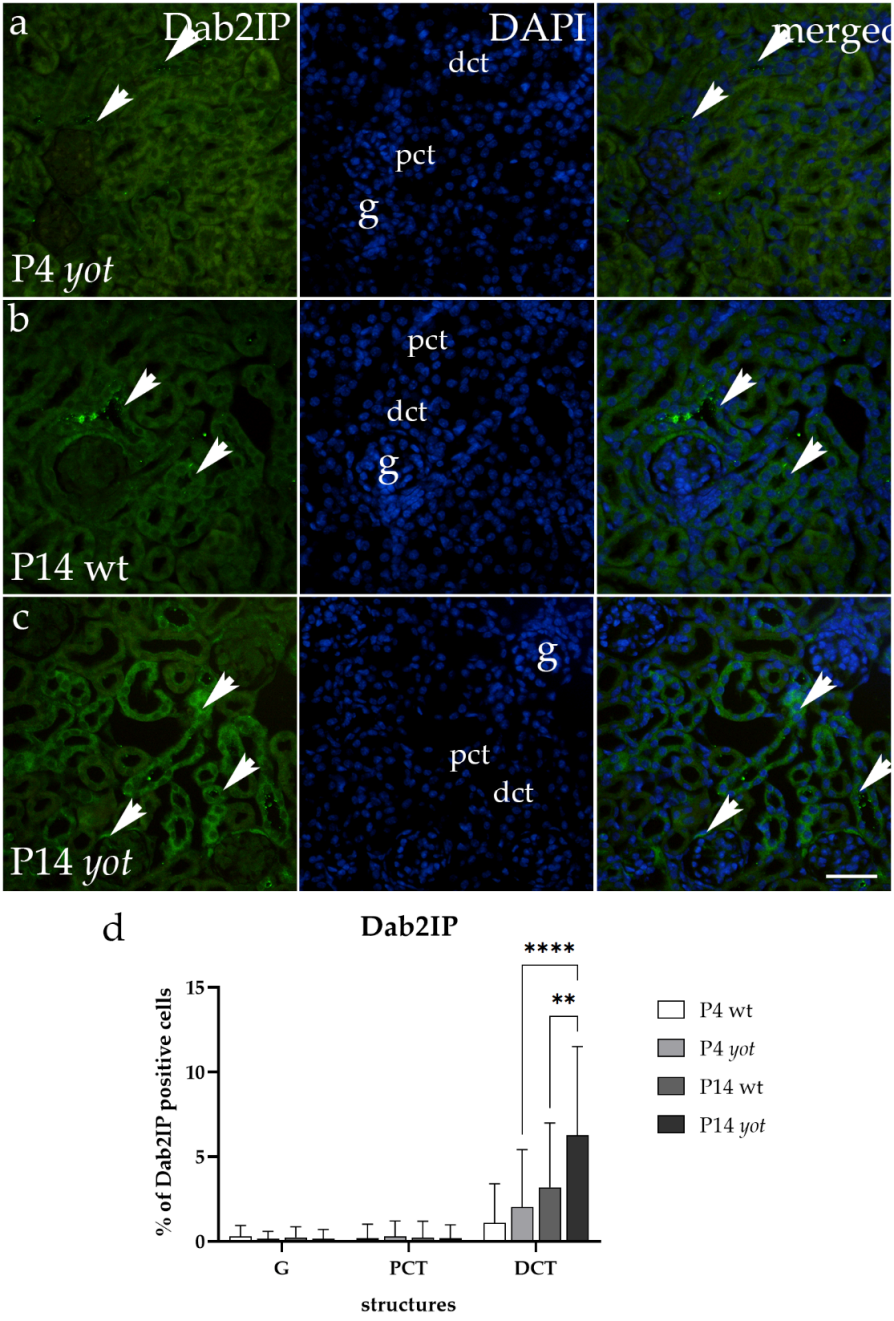
Immunofluorescence staining of postnatal wild-type and *yotari* mouse kidneys was performed using the Dab2IP (**a**–**c**) markers. Arrows show the immunoexpression patterns of Dab2IP in glomeruli (g), distal convoluted tubules (dct) and proximal convoluted tubules (pct), indicated on 4′,6-diamidino-2-phenylindole (DAPI) nuclei staining image. Immunoexpression of Dab2IP staining and merged Dab2IP and DAPI at 4 days (P4) and 14 days (P14) in wild type (**b**) and *yotari* (**a**,**c**) mice. Wild-type and *yotari* mice at postnatal day 4 of kidney development predominantly matched the localization and intensity of Caveolin-1. Therefore, representative images were taken from wild-type mice. Images were taken at ×40 magnification, and the scale bar represents 100 μm for all images. The percentages of Dab2IP positive cells (**d**) in the glomeruli (G), distal convoluted tubules (DCT) and proximal convoluted tubules (PCT) of postnatal kidneys of wild type and *yotari* animals over time (P4 and P14). Data are presented as the mean ± SD (vertical line) and analyzed using a two-way ANOVA followed by Tukey’s multiple comparison test. Significant differences are indicated by ** *p* < 0.01, **** *p* < 0.0001. Twenty substructures were assessed at each time point.

Postnatally, Dab2IP was observed as punctuative staining in distal convoluted tubules and the parietal layer of the Bowman’s capsule (Figure 8a–c and Figure S10). The percentage of Dab2IP positive cells was highest at P14 in both wt and *yot* mice, and there was a statistically significant difference in the percentage of Dab2IP positive cells in distal convoluted tubules at P4 and P14 of wt and *yot* mice (*p* < 0.00001 and *p* < 0.01, respectively) (Figure 8d). The staining intensity Dab2IP was mild in the distal convoluted tubules in both phenotypes in all observed stages (Table 3), while there was no reactivity in other structures.

## 4. Discussion

There are a number of different proteins that transfer signal information to the cells. More precisely, they act as ligands that bind to receptors immunoexpressed on their target cells [35]. Therefore, in this study, we wanted to explore some of those signal markers during kidney development and their potential function in CAKUT. CAKUT is a spectrum of embryonic disorders in the kidney and outflow tract [36]. Many genes and mutations have been implicated in the genesis of these conditions. Previous studies with *yotari* mice found different markers and how disruption of signaling tracking can cause CAKUT [1,9,10,37].

In our study, we used a *Dab1*^−/−^ mouse model, and we hypothesized that expression of endocytic receptors such as Megalin, Cubilin, Caveolin-1, Gipc1 and Dab2IP have different patterns at embryonic and postnatal developmental periods.

Megalin and Cubilin displayed distinct immunoexpression patterns in different stages of wt and *yot* kidney development within the renal structures of interest. In the study by De et al., Megalin was found to be immunoexpressed at the renal proximal tubule brush border, the membrane recycling system and the endocytic compartments [38]. Megalin can be found in lysosomes, but after endocytosis, dense apical tubules return the main quantities of Megalin to the apical membrane [13,39]. Similarly, our study observed high immunoexpression of Megalin in the ampulla/collecting ducts and convoluted tubules across all examined stages in the embryonic period and the proximal convoluted tubules at the postnatal phases. Additionally, we observed Megalin immunoexpression in the parietal layer of the Bowman’s capsule and the glomeruli. The research of Prabakaran et al. showed that the immunoexpression of Megalin in the podocytes has been identified in rats and humans [40]. All these findings suggest that this protein plays a significant role in these renal structures throughout development and indicates its involvement in the key physiological processes within kidneys.

Interestingly, our study found a decreased percentage of Megalin-positive cells in the ampulla/collecting ducts and convoluted tubules of *yot* mice compared to the wt mice at E13.5. This finding is in line with the study by Motoyoshi et al., which documented that glomerular injury is imposed in mice with reduced immunoexpression of Megalin [41]. In the postnatal period, the percentage of positive Megalin cells was highest at P4 in both wt and *yot* mice and decreased in the later developmental stage (P14). This finding suggests the importance of Megalin during nephrogenesis and proper nephron differentiation. It is especially important for the endocytic process within proximal convoluted tubules since the loss of such Megalin can contribute to proteinuria development, an important clinical indicator of kidney diseases [38]. Additionally, Megalin plays an important role in the endocytosis of different ligands, which mediate the trafficking of Megalin in proximal tubular epithelial cells. This might be a potential therapeutical target for drug-caused nephrotoxicity or metabolic kidney disease [13].

Cubilin immunoexpression was higher in wt mice compared to *yot* at E13.5 while at E15.5. Cubilin immunoexpressions were low in both wt and *yot*. This might suggest a complex interplay between Cubilin immunoexpression and developmental stages, potentially indicating the need for compensatory mechanisms in *yot* mice with deficient immunoexpression of Cubilin at E13.5 compared to wt. In the postnatal developmental stages, Cubilin staining was most prominent at the apical membranes of cells of proximal convoluted tubules. This finding aligns with Cubilin’s role in the kidney since it is a crucial protein needed for tubular protein reabsorption, including albumin [39]. Namely, Cubilin is abundantly immunoexpressed at the proximal tubule and binds with Megalin, consequently increasing their multiligand capability. Therefore, the co-expression of Megalin and Cubilin at the apical membrane of convoluted tubules suggests their importance in reabsorption and proper nephron function. Namely, it has been demonstrated that Cubilin interacts with different intracellular proteins for trafficking as well as with other membrane molecules, such as the Cubilin–Amnionless complex [38].

We noticed a high immunoexpression of Caveolin-1 in the metanephric mesenchyme, blood vessels and the border area between the metanephric mesenchyme and renal vesicle across all examined stages of development. This widespread immunoexpression pattern suggests that Caveolin-1 may play pivotal roles in various processes crucial for kidney development, including mesenchymal–epithelial transition, vascularization and cell–cell interactions within the nephrogenic niche. Interestingly, the percentage of positive cells with Caveolin-1 in the metanephric mesenchyme was significantly higher in wt mice compared to *yot* mice at both E13.5 and E15.5, indicating potential differences in Caveolin-1 regulation between the two phenotypes during early kidney development. Additionally, the immunoexpression of Caveolin-1 decreased with developmental progression, suggesting a dynamic regulation of Caveolin-1 immunoexpression during kidney organogenesis. Our results align with the study by Luo et al., who found that Caveolin-1 has an important part in cellular metabolism during autophagy. Namely, autophagy is closely related to the metabolism and survival of cells, which is important for proper nephrogenesis [37,42]. Furthermore, Caveolin-1 staining was most prominent in the blood vessels, glomeruli and the outer layer of the Bowman’s capsule in postnatal developmental stages. This distribution pattern suggests potential roles for Caveolin-1 in cellular biological activities, including cell metabolism and growth. The highest percentage of Caveolin-1 positive cells was observed at P14 in both wt and *yot* mice, with a statistically significant difference between the two phenotypes at this stage. This finding implies potential implications of Caveolin-1 dysregulation in the context of postnatal kidney development and function. Namely, Caveolin-1 may have a possible role in the pathophysiology of acute kidney injury by regulating cellular metabolism and activities regarding cellular life [27]. These findings were used to develop Caveolin-1 targeted treatments of different diseases, including kidney diseases. Moreover, the staining intensity of Caveolin-1 was strongest in the glomeruli of both phenotypes at all examined stages, suggesting a specific role for Caveolin-1 in glomerular physiology. Our results align with the research on glomerulonephritis that showed consistent changes in the kidney transcriptome, which may indicate how abnormal cellular metabolism plays an important role in this disease [43]. The absence of Caveolin-1 staining in other structures in our study suggests a selective immunoexpression pattern of Caveolin-1 within the kidney.

Gipc1 exhibited diffuse cytoplasmic staining during renal development in the metanephric mesenchyme, convoluted tubules and collecting ducts. This widespread distribution suggests the potential involvement of Gipc1 in various cellular processes within these renal structures, such as cell signaling, cytoskeletal organization and intracellular trafficking. GIPC is a scaffold that organizes receptor-mediated trafficking, and after receptor internalization, it connects with endocytic vesicles near the plasma membrane [44]. Interestingly, the percentage of Gipc1 positive cells at E13.5 was highest in *yot* mice compared to wt mice in the metanephric mesenchyme, indicating potential differences in Gipc1 regulation between the two phenotypes during early kidney development. However, by E15.5, the percentage of Gipc1 positive cells was highest in *yot* mice in the ampulla/collecting ducts compared to wt mice, suggesting compensatory changes in Gipc1 immunoexpression at later stages. Furthermore, an increase in the number of Gipc1 positive cells was observed in convoluted tubules of wt mice compared to *yot* mice at E15.5, indicating potential dynamic differences in Gipc1 immunoexpression within specific renal structures during development. The staining intensity of Gipc1 was strongest in the metanephric mesenchyme in both phenotypes at E13.5, suggesting potential roles for Gipc1 in the early renal development processes occurring within this structure. In later developmental stages, moderate staining reactivity was noticed in convoluted tubules of wt mice and ampulla/collecting ducts of *yot* mice, indicating potential changes in Gipc1 immunoexpression and distribution as development progresses. During the postnatal period, Gipc1 was observed as punctuated signals in the cytoplasm of all observed structures. Lou et al. showed that GIPC can be found in clathrin-coated pits as well as at apical tubules of endocytic compartments in the renal brush border [45]. The occurrence and interaction of GIPC and Megalin in the endosomal compartments of the proximal tubules suggest that it can be a model that regulates Megalin’s endocytic function [45].

DAB2IP has been implicated in the regulation of a diverse array of biological processes, including proliferation [46] and epithelial-to-mesenchymal transition [47]. Our results showed that the immunoexpression pattern of Dab2IP varied during renal development and postnatal stages between wt and *yot* mice. At E13.5, Dab2IP staining was prominently observed in the renal vesicle/glomeruli and ampulla/collecting ducts in wt mice, whereas in *yot* mice, staining was predominantly noticed in convoluted tubules and metanephric mesenchyme. This indicates a differential localization of Dab2IP in these developmental stages due to the delayed immunoexpression of Dab2IP in *yot* mice in mesenchymal structures that are precursors of the future nephrons. Furthermore, at E15.5, there was an absence of Dab2IP reactivity in all structures and stages, indicating a stable regulation of Dab2IP immunoexpression during this phase. In the postnatal stages, Dab2IP staining was observed as punctuative staining in distal convoluted tubules and the parietal layer of the Bowman’s capsule. The highest percentage of Dab2IP positive cells was observed at P14 in both wt and *yot* mice, with significant differences between P4 and P14 in both phenotypes and almost double the immunoexpression percentage in *yot* mice. This suggests a delayed regulation of Dab2IP immunoexpression postnatally due to Dab2IP involvement in the signaling of mitogen-activated protein kinases. Namely, Dab2IP has been shown to regulate the RAS-ERK signaling pathway, where depletion of Dab2IP leads to MAPK pathway activation that responds to proliferation, differentiation, and apoptosis necessary for final kidney nephrogenesis [10,48,49]. Therefore, high immunoexpression of Dab2IP in *yot* mice in postnatal stages might imply deregulated survival and apoptosis pathways.

The primary limitation of our study is its observational design. We did not perform quantitative protein immunoexpression analyses like flow cytometry or Western blotting. Another limitation of our study is that we did not conduct Western blot analyses or validate the antibodies in cell cultures to confirm their specificity.

## 5. Conclusions

Our results provide valuable insights into the spatiotemporal immunoexpression pattern and potential roles of Megalin, Cubilin, Caveolin-1, Gipc1 and Dab2IP in renal development and postnatal maturation of wt and *yot* mice, highlighting the dynamic nature of their regulation and potential implications for renal pathology in loss of Dab1 function. These findings contribute to understanding the molecular mechanisms underlying kidney development and CAKUT, providing valuable insights into potential therapeutic targets and diagnostic markers for renal diseases and developmental abnormalities. Further studies elucidating these proteins’ precise roles and interactions could contribute to a better understanding of kidney development and may offer therapeutic targets for renal disorders. 

## Figures and Tables

**Table 1:** Antibodies used for immunofluorescence.

Antibodies		Catalog Number	Host	Dilution	Source
Primary	Anti-Lrp2/Megalin antibody	ab76969	Rabbit	1:250	Abcam, Cambridge, UK
	Human/Mouse/Rat Cubilin Antibody	#AF3700	Sheep	1:50	R&D Systems, Inc., Minneapolis, MN, USA
	Caveolin-1 (D46G3) XP^®^ Rabbit mAb	#3267S	Rabbit	1:300	Cell Signaling Technology (CST), Danvers, MA, USA
	Gipc1 Polyclonal antibody	14822-1-AP	Rabbit	1:100	Proteintech Group, Inc., Rosemont, IL, USA
	Dab2IP Polyclonal antibody	23582-1-AP	Rabbit	1:50	Proteintech Group, Inc., Rosemont, IL, USA
	Anti-Aquaporin 2/AQP2 (E-2)	sc-515770	Mouse	1:50	Santa Cruz Biotechnology Inc., Santa Cruz, CA, USA
Lectins	Fluorescein labeled Lotus Tetragonolobus lectin (LTL)	FL-1321	N/A	1:400	Vector Laboratories Ltd., Peterborough, UK
	Fluorescein labeled Dolichos biflorus agglutinin (DBA)	FL-1031	N/A	1:400	Vector Laboratories Ltd., Peterborough, UK
Secondary	Alexa Fluor^®^ 488 AffiniPure™ Donkey Anti-Rabbit IgG (H + L)	711-545-152	Donkey	1:300	Jackson Immuno Research Laboratories, Inc., Baltimore, PA, USA
	Rhodamine Red™-X (RRX) AffiniPure™ Donkey Anti-Rabbit IgG (H + L)	711-295-152	Donkey	1:300	Jackson Immuno Research Laboratories, Inc., Baltimore, PA, USA
	Alexa Fluor^®^ 488 AffiniPure™ Donkey Anti-Mouse IgG (H + L)	715-545-150	Donkey	1:300	Jackson Immuno Research Laboratories, Inc., Baltimore, PA, USA
	Rhodamine Red™-X (RRX) AffiniPure™ Donkey Anti-Sheep IgG (H + L)	713-295-003	Donkey	1:300	Jackson Immuno Research Laboratories, Inc., Baltimore, PA, USA

**Table 2:** Staining intensity of specific antibodies in the kidneys of *yotari* and wild-type mice at embryonic days E13.5 and E15.5.

Embryonic Day (E)	Animal	Structure	Megalin	Cubilin	Caveolin	Dab2IP	GIPC-1
E13.5	wild type	mm	+	+++	+++	+	++
		rv/g	+	+	−	+	+
		Ct	++	+	−	+	+
		A/Cd	+++	+	−	+	+
	*yotari*	mm	+	+	++	+	+++
		rv/g	++	+	−	−	−
		Ct	++	+	−	+	+
		A/Cd	++	+	−	+	+
E15.5	wild type	mm	+	+	++	−	−
		rv/g	+	+	+	−	−
		Ct	+++	+	+	+	++
		A/Cd	+	+	+	+	−
	*yotari*	mm	+	+	+	−	−
		rv/g	+	+	+	−	−
		Ct	+	+++	+	−	−
		A/Cd	+++	+	+	−	++

+++ strong reactivity; ++ moderate reactivity; + mild reactivity; − no reactivity; mm—metanephric mesenchyme, rv—renal vesicle, g—immature glomeruli, Ct—convoluted tubule, A—ampulla, Cd—collecting duct, E—day of embryonic development.

**Table 3:** Staining intensity of specific antibodies in the kidneys of *yotari* and wild-type mice at postnatal days P4, P11 and P14.

Postnatal Day (P)	Animal	Structure	Megalin	Cubilin	Caveolin	Dab2IP	GIPC
P4	wild type	g	−	−	+	−	+
		pct	+++	+++	−	−	−
		dct	+	+	−	+	+
	*yotari*	g	−	−	+	+	−
		pct	+++	+++	−	+	+
		dct	+	+	−	+	+
P14	wild type	g	−	−	+	−	−
		pct	+++	+++	−	−	+
		dct	+	+	−	+	+
	*yotari*	g	−	−	+	−	−
		pct	+++	+++	−	−	−
		dct	−	−	+	+	+

+++ strong reactivity; + mild reactivity; − no reactivity; g—glomeruli, pct—proximal convoluted tubules, dct—distal convoluted tubules, p—day of postnatal development.

## Data Availability

All data and materials are available upon request due to (specify the reason for the restriction).

## Supplementary Materials

The following supporting information can be downloaded at: https://www.mdpi.com/article/10.3390/biomedicines12071542/s1, Figure S1. Double immunofluorescence staining of embryonic day 15.5 (E15.5) *yotari* (*yot*) mouse kidneys of the *Lotus tetragonolobus* lectin (LTL), a marker for proximal tubules (a), *Dolichos biflorus* agglutinin (DBA), a marker for developing renal collecting ducts (b), and Aquaporin 2 (AQP2), a marker for collecting ducts (c) with Megalin (a–c) marker. Arrows show the expression pattern of LTL, DBA, AQP2 and Megalin in metanephric mesenchyme (mm), renal vesicles (rv), glomeruli (g), convoluted tubules (ct), ampullae (A), and collecting ducts (Cd) indicated on 4′,6-diamidino-2-phenylindole (DAPI) nuclei staining image. An asterisk on merged LTL, DBA, AQP2, Megalin and DAPI image denotes the substructure where co-expression was detected. Images were taken on magnification ×40. The scale bar is 50 μm, which refers to all images; Figure S2. Double immunofluorescence staining of postnatal day 4 (P4) *yotari* (*yot*) mouse kidney cortex of the *Lotus tetragonolobus* lectin (LTL), a marker for proximal tubules (a), *Dolichos biflorus* agglutinin (DBA), a marker for distal tubules (b), and Aquaporin 2 (AQP2), a marker for distal and collecting tubules (c) with Megalin (a–c) marker. Arrows show the expression pattern of LTL, DBA, AQP2 and Megalin in glomeruli (g), proximal convoluted tubules (pct), distal convoluted tubules (dct) and collecting tubules (Ct) indicated on 4′,6-diamidino-2-phenylindole (DAPI) nuclei staining image. An asterisk on merged LTL, DBA, AQP2, Megalin and DAPI image denotes the substructure where co-expression was detected. Images were taken on magnification ×40. The scale bar is 50 μm, which refers to all images; Figure S3. Double immunofluorescence staining of embryonic day 15.5 (E15.5) *yotari* (*yot*) mouse kidneys of the *Lotus tetragonolobus* lectin (LTL), a marker for proximal tubules (a), *Dolichos biflorus* agglutinin (DBA), a marker for developing renal collecting ducts (b), and Aquaporin 2 (AQP2), a marker for collecting ducts (c) with Cubilin (a–c) marker. Arrows show the expression pattern of LTL, DBA, AQP2 and Cubilin in metanephric mesenchyme (mm), renal vesicles (rv), glomeruli (g), convoluted tubules (ct), ampullae (A), and collecting ducts (Cd) indicated on 4′,6-diamidino-2-phenylindole (DAPI) nuclei staining image. An asterisk on merged LTL, DBA, AQP2, Cubilin and DAPI image denotes the substructure where co-expression was detected. Images were taken on magnification ×40. The scale bar is 50 μm, which refers to all images; Figure S4. Double immunofluorescence staining of postnatal day 4 (P4) *yotari* (*yot*) mouse kidney cortex of the *Lotus tetragonolobus* lectin (LTL), a marker for proximal tubules (a), *Dolichos biflorus* agglutinin (DBA), a marker for distal tubules (b), and Aquaporin 2 (AQP2), a marker for distal and collecting tubules (c) with Cubilin (a–c) marker. Arrows show the expression pattern of LTL, DBA, AQP2 and Cubilin in glomeruli (g), proximal convoluted tubules (pct), distal convoluted tubules (dct) and collecting tubules (Ct) indicated on 4′,6-diamidino-2-phenylindole (DAPI) nuclei staining image. An asterisk on merged LTL, DBA, AQP2, Cubilin and DAPI image denotes the substructure where co-expression was detected. Images were taken on magnification ×40. The scale bar is 50 μm, which refers to all images; Figure S5. Double immunofluorescence staining of embryonic day 15.5 (E15.5) *yotari* (*yot*) mouse kidneys of the *Lotus tetragonolobus* lectin (LTL), a marker for proximal tubules (a), *Dolichos biflorus* agglutinin (DBA), a marker for developing renal collecting ducts (b), and Aquaporin 2 (AQP2), a marker for collecting ducts (c) with Caveolin-1 (a–c) marker. Arrows show the expression pattern of LTL, DBA, AQP2 and Caveolin-1 in metanephric mesenchyme (mm), renal vesicles (rv), glomeruli (g), convoluted tubules (ct), ampullae (A), and collecting ducts (Cd) indicated on 4′,6-diamidino-2-phenylindole (DAPI) nuclei staining image. An asterisk on merged LTL, DBA, AQP2, Caveolin-1 and DAPI image denotes the substructure where co-expression was detected. Images were taken on magnification ×40. The scale bar is 50 μm, which refers to all images; Figure S6. Double immunofluorescence staining of postnatal day 4 (P4) *yotari* (*yot*) mouse kidney cortex of the *Lotus tetragonolobus* lectin (LTL), a marker for proximal tubules (a), *Dolichos biflorus* agglutinin (DBA), a marker for distal tubules (b), and Aquaporin 2 (AQP2), a marker for distal and collecting tubules (c) with Caveolin-1 (a–c) marker. Arrows show the expression pattern of LTL, DBA, AQP2 and Caveolin-1 in glomeruli (g), proximal convoluted tubules (pct), distal convoluted tubules (dct), blood vessels (bv) and collecting tubules (Ct) indicated on 4′,6-diamidino-2-phenylindole (DAPI) nuclei staining image. An asterisk on merged LTL, DBA, AQP2, Caveolin-1 and DAPI image denotes the substructure where co-expression was detected. Images were taken on magnification ×40. The scale bar is 50 μm, which refers to all images; Figure S7. Double immunofluorescence staining of embryonic day 15.5 (E15.5) *yotari* (*yot*) mouse kidneys of the *Lotus tetragonolobus* lectin (LTL), a marker for proximal tubules (a), *Dolichos biflorus* agglutinin (DBA), a marker for developing renal collecting ducts (b), and Aquaporin 2 (AQP2), a marker for collecting ducts (c) with Gipc-1 (a–c) marker. Arrows show the expression pattern of LTL, DBA, AQP2 and Gipc-1 in metanephric mesenchyme (mm), renal vesicles (rv), glomeruli (g), convoluted tubules (ct), ampullae (A), and collecting ducts (Cd) indicated on 4′,6-diamidino-2-phenylindole (DAPI) nuclei staining image. An asterisk on merged LTL, DBA, AQP2, Gipc-1 and DAPI image denotes the substructure where co-expression was detected. Images were taken on magnification ×40. The scale bar is 50 μm, which refers to all images; Figure S8. Double immunofluorescence staining of postnatal day 4 (P4) *yotari* (*yot*) mouse kidney cortex of the *Lotus tetragonolobus* lectin (LTL), a marker for proximal tubules (a), *Dolichos biflorus* agglutinin (DBA), a marker for distal tubules (b), and Aquaporin 2 (AQP2), a marker for distal and collecting tubules (c) with Gipc-1 (a–c) marker. Arrows show the expression pattern of LTL, DBA, AQP2 and Gipc-1 in glomeruli (g), proximal convoluted tubules (pct), distal convoluted tubules (dct) and collecting tubules (Ct) indicated on 4′,6-diamidino-2-phenylindole (DAPI) nuclei staining image. An asterisk on merged LTL, DBA, AQP2, Gipc-1 and DAPI image denotes the substructure where co-expression was detected. Images were taken on magnification ×40. The scale bar is 50 μm, which refers to all images; Figure S9. Double immunofluorescence staining of embryonic day 15.5 (E15.5) *yotari* (*yot*) mouse kidneys of the *Lotus tetragonolobus* lectin (LTL), a marker for proximal tubules (a), *Dolichos biflorus* agglutinin (DBA), a marker for developing renal collecting ducts (b), and Aquaporin 2 (AQP2), a marker for collecting ducts (c) with Dab2IP (a–c) marker. Arrows show the expression pattern of LTL, DBA, AQP2 and Dab2IP in metanephric mesenchyme (mm), renal vesicles (rv), glomeruli (g), convoluted tubules (ct), ampullae (A), and collecting ducts (Cd) indicated on 4′,6-diamidino-2-phenylindole (DAPI) nuclei staining image. An asterisk on merged LTL, DBA, AQP2, Dab2IP and DAPI image denotes the substructure where co-expression was detected. Images were taken on magnification ×40. The scale bar is 50 μm, which refers to all images; Figure S10. Double immunofluorescence staining of postnatal day 4 (P4) *yotari* (*yot*) mouse kidney cortex of the *Lotus tetragonolobus* lectin (LTL), a marker for proximal tubules (a), *Dolichos biflorus* agglutinin (DBA), a marker for distal tubules (b), and Aquaporin 2 (AQP2), a marker for distal and collecting tubules (c) with Dab2IP (a–c) marker. Arrows show the expression pattern of LTL, DBA, AQP2 and Dab2IP in glomeruli (g), proximal convoluted tubules (pct), distal convoluted tubules (dct) and collecting tubules (Ct) indicated on 4′,6-diamidino-2-phenylindole (DAPI) nuclei staining image. An asterisk on merged LTL, DBA, AQP2, Dab2IP and DAPI image denotes the substructure where co-expression was detected. Images were taken on magnification ×40. The scale bar is 50 μm, which refers to all images; Figure S11. Negative control staining images (primary antibodies were omitted from the immunofluorescent protocol and only secondary antibody were applied on the sections) of embryonic day 15.5 (E15.5) *yotari* (*yot*) mouse kidneys and postnatal day 4 (P4) *yotari* (*yot*) mouse kidney cortex. Images were taken on magnification ×40. The scale bar is 50 μm, which refers to all images.
