# Supplementary material for: Immunoexpression Patterns of Megalin, Cubilin, Caveolin-1, Gipc1 and Dab2IP in the Embryonic and Postnatal Development of the Kidneys in Yotari (Dab1−/−) Mice
Source: Biomedicines. 2024 Jul 11;12(7):1542. doi: 10.3390/biomedicines12071542 (PMC11274389; doi:10.3390/biomedicines12071542)
Supplement: Supplementary file 1 [file biomedicines-12-01542-s001.zip › biomedicines-3084607-Supplementary.pdf]

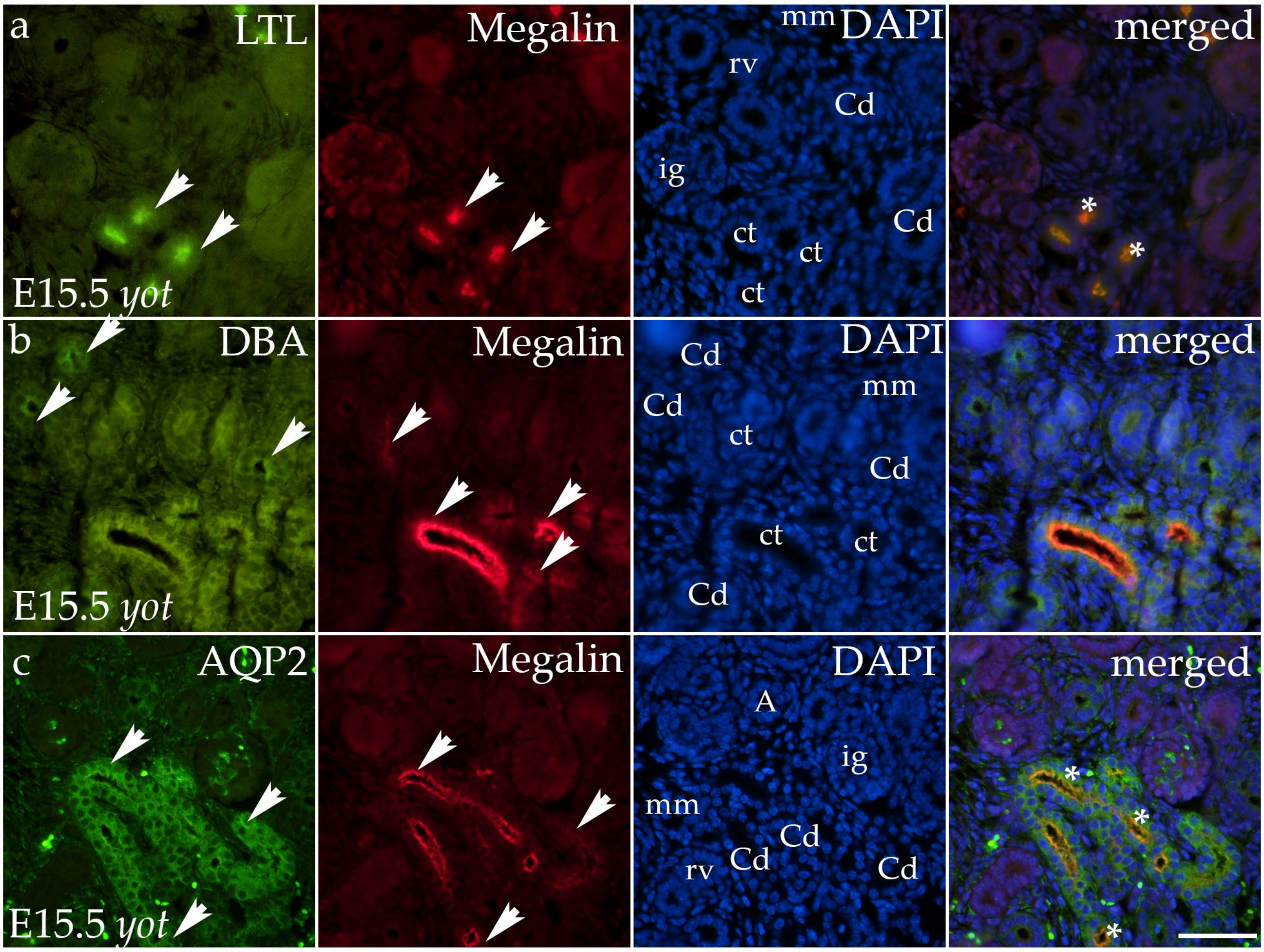

Figure S1. Double immunofluorescence staining of embryonic day 15.5 (E15.5) *yotari* (*yot*) mouse kidneys of the *Lotus tetragonolobus* lectin (LTL), a marker for proximal tubules (**a**), *Dolichos biflorus* agglutinin (DBA), a marker for developing renal collecting ducts (**b**), and Aquaporin 2 (AQP2), a marker for collecting ducts (**c**) with Megalin (**a**–**c**) marker. Arrows show the expression pattern of LTL, DBA, AQP2 and Megalin in metanephric mesenchyme (mm), renal vesicles (rv), glomeruli (g), convoluted tubules (ct), ampullae (A), and collecting ducts (Cd) indicated on 4′,6-diamidino-2-phenylindole (DAPI) nuclei staining image. An asterisk on merged LTL, DBA, AQP2, Megalin and DAPI image denotes the substructure where co-expression was detected. Images were taken on magnification ×40. The scale bar is 50 μm, which refers to all images.

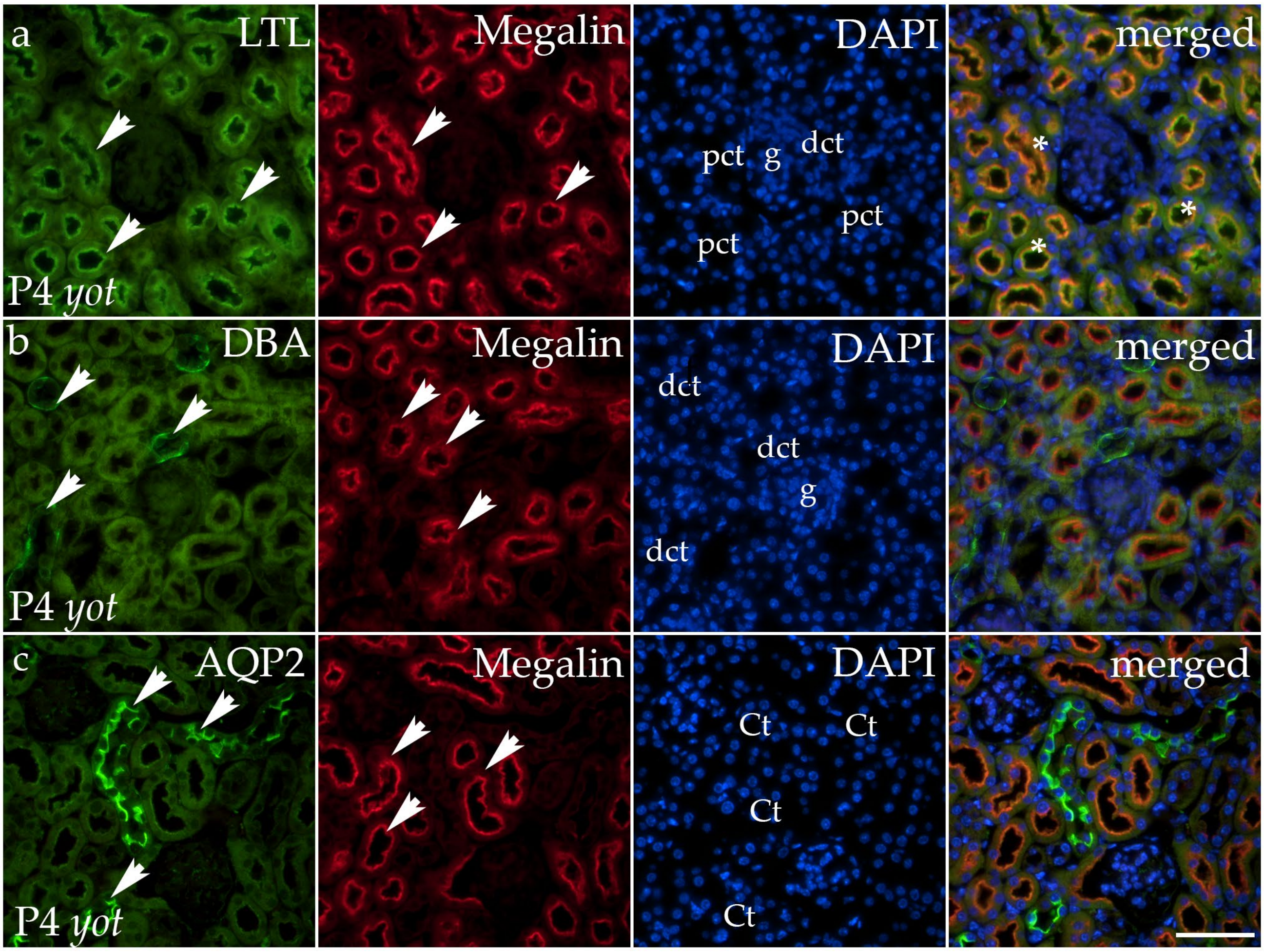

Figure S2. Double immunofluorescence staining of postnatal day 4 (P4) *yotari* (*yot*) mouse kidney cortex of the *Lotus tetragonolobus* lectin (LTL), a marker for proximal tubules (**a**), *Dolichos biflorus* agglutinin (DBA), a marker for distal tubules (**b**), and Aquaporin 2 (AQP2), a marker for distal and collecting tubules (**c**) with Megalin (**a**–**c**) marker. Arrows show the expression pattern of LTL, DBA, AQP2 and Megalin in glomeruli (g), proximal convoluted tubules (pct), distal convoluted tubules (dct) and collecting tubules (Ct) indicated on 4′,6-diamidino-2-phenylindole (DAPI) nuclei staining image. An asterisk on merged LTL, DBA, AQP2, Megalin and DAPI image denotes the substructure where co-expression was detected. Images were taken on magnification ×40. The scale bar is 50 μm, which refers to all images.

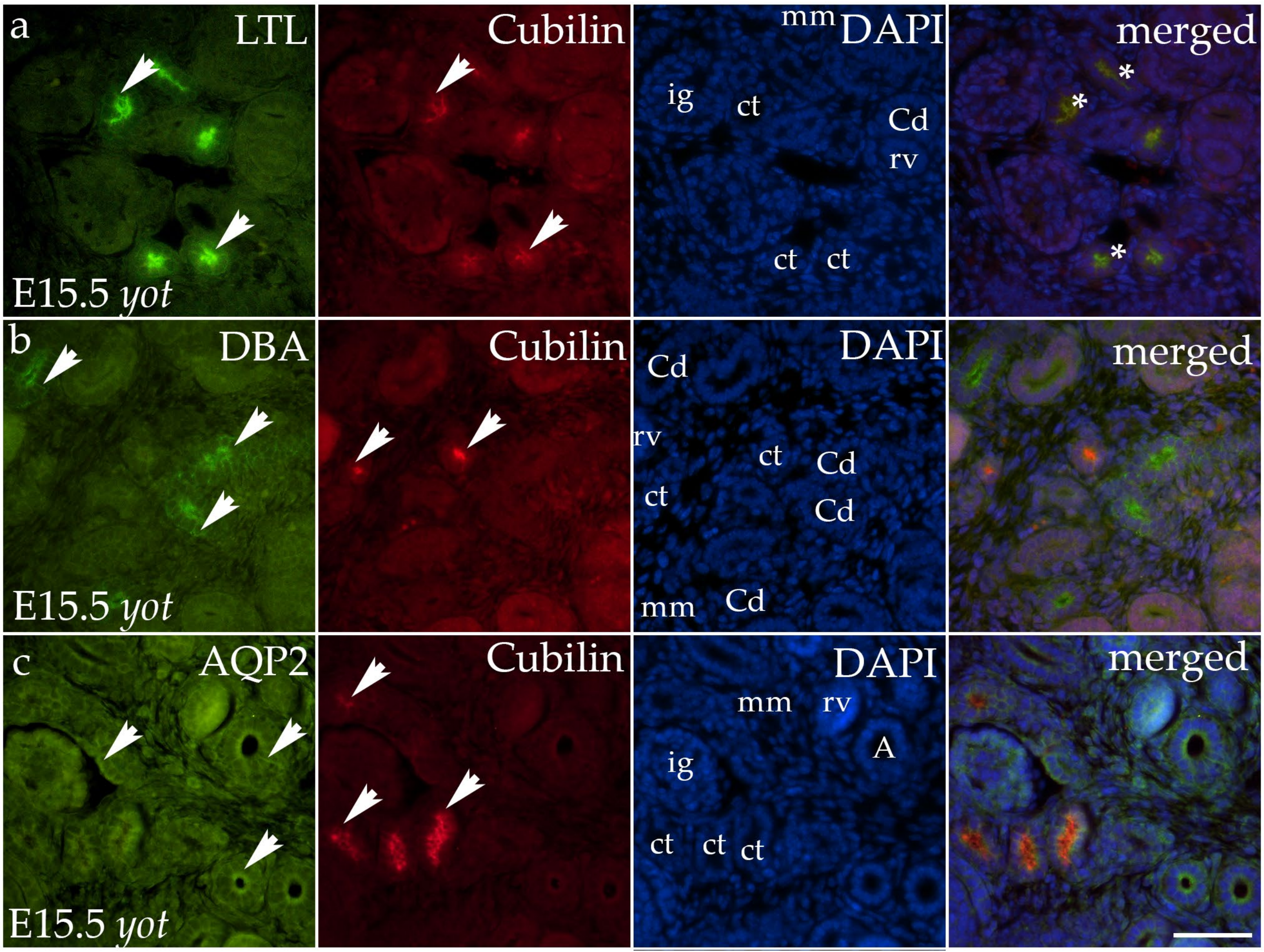

Figure S3. Double immunofluorescence staining of embryonic day 15.5 (E15.5) *yotari* (*yot*) mouse kidneys of the *Lotus tetragonolobus* lectin (LTL), a marker for proximal tubules (**a**), *Dolichos biflorus* agglutinin (DBA), a marker for developing renal collecting ducts (**b**), and Aquaporin 2 (AQP2), a marker for collecting ducts (**c**) with Cubilin (**a**–**c**) marker. Arrows show the expression pattern of LTL, DBA, AQP2 and Cubilin in metanephric mesenchyme (mm), renal vesicles (rv), glomeruli (g), convoluted tubules (ct), ampullae (A), and collecting ducts (Cd) indicated on 4′,6-diamidino-2-phenylindole (DAPI) nuclei staining image. An asterisk on merged LTL, DBA, AQP2, Cubilin and DAPI image denotes the substructure where co-expression was detected. Images were taken on magnification ×40. The scale bar is 50 μm, which refers to all images.

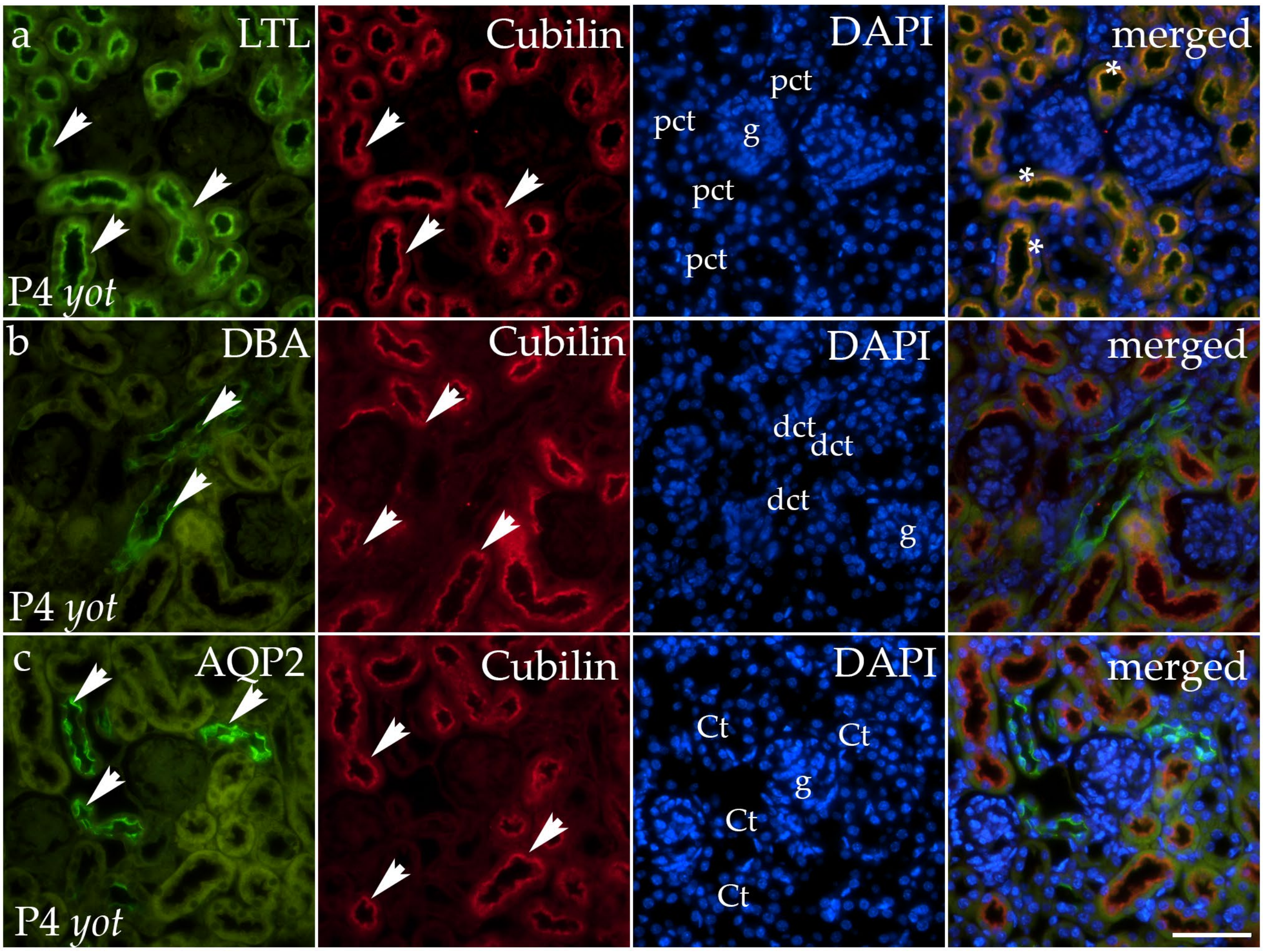

Figure S4. Double immunofluorescence staining of postnatal day 4 (P4) *yotari* (*yot*) mouse kidney cortex of the *Lotus tetragonolobus* lectin (LTL), a marker for proximal tubules (**a**), *Dolichos biflorus* agglutinin (DBA), a marker for distal tubules (**b**), and Aquaporin 2 (AQP2), a marker for distal and collecting tubules (**c**) with Cubilin (**a**–**c**) marker. Arrows show the expression pattern of LTL, DBA, AQP2 and Cubilin in glomeruli (g), proximal convoluted tubules (pct), distal convoluted tubules (dct) and collecting tubules (Ct) indicated on 4′,6-diamidino-2-phenylindole (DAPI) nuclei staining image. An asterisk on merged LTL, DBA, AQP2, Cubilin and DAPI image denotes the substructure where co-expression was detected. Images were taken on magnification ×40. The scale bar is 50 µm, which refers to all images.

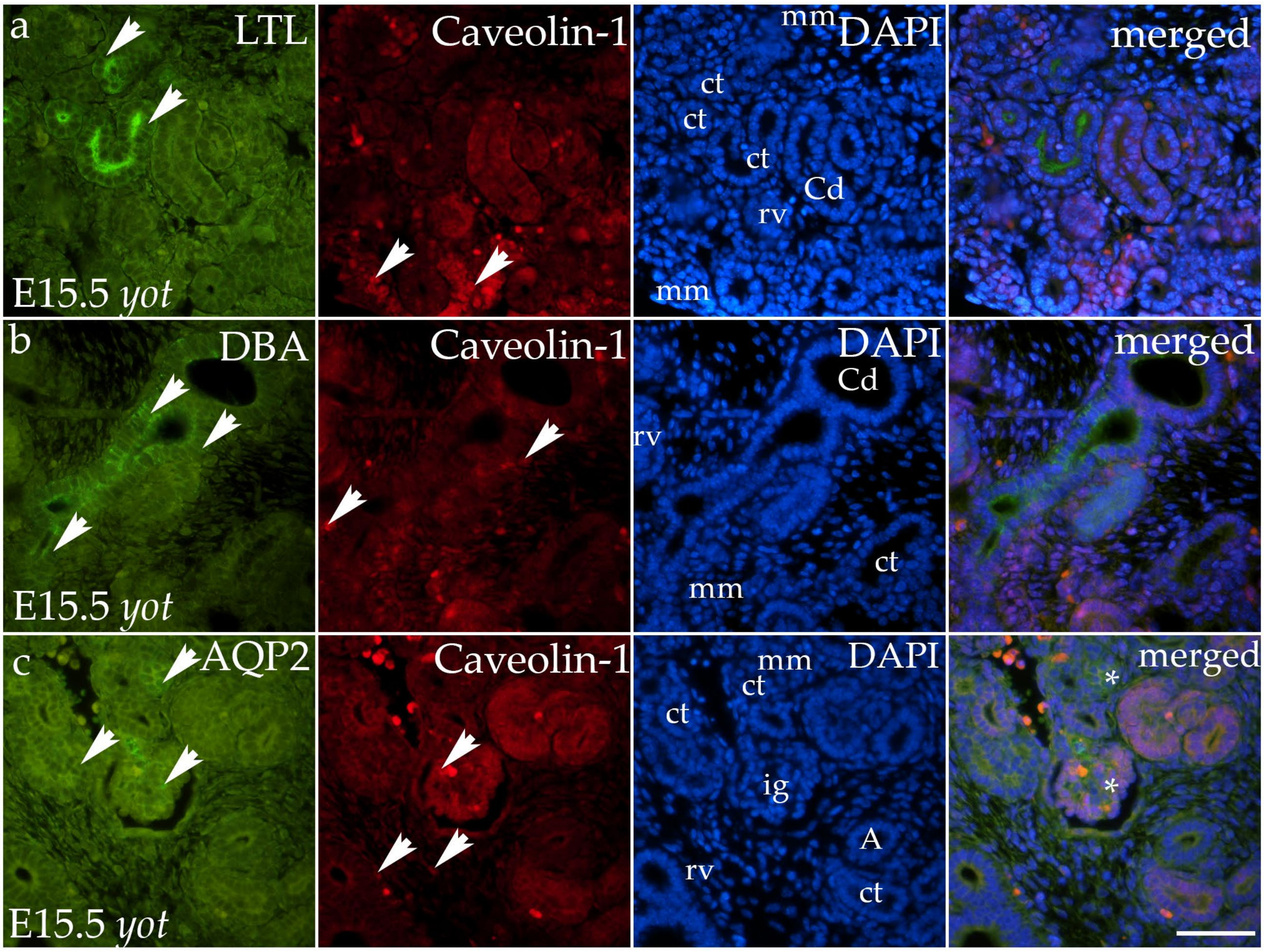

Figure S5. Double immunofluorescence staining of embryonic day 15.5 (E15.5) *yotari* (*yot*) mouse kidneys of the *Lotus tetragonolobus* lectin (LTL), a marker for proximal tubules (**a**), *Dolichos biflorus* agglutinin (DBA), a marker for developing renal collecting ducts (**b**), and Aquaporin 2 (AQP2), a marker for collecting ducts (**c**) with Caveolin-1 (**a**–**c**) marker. Arrows show the expression pattern of LTL, DBA, AQP2 and Caveolin-1 in metanephric mesenchyme (mm), renal vesicles (rv), glomeruli (g), convoluted tubules (ct), ampullae (A), and collecting ducts (Cd) indicated on 4′,6-diamidino-2-phenylindole (DAPI) nuclei staining image. An asterisk on merged LTL, DBA, AQP2, Caveolin-1 and DAPI image denotes the substructure where co-expression was detected. Images were taken on magnification ×40. The scale bar is 50 μm, which refers to all images.

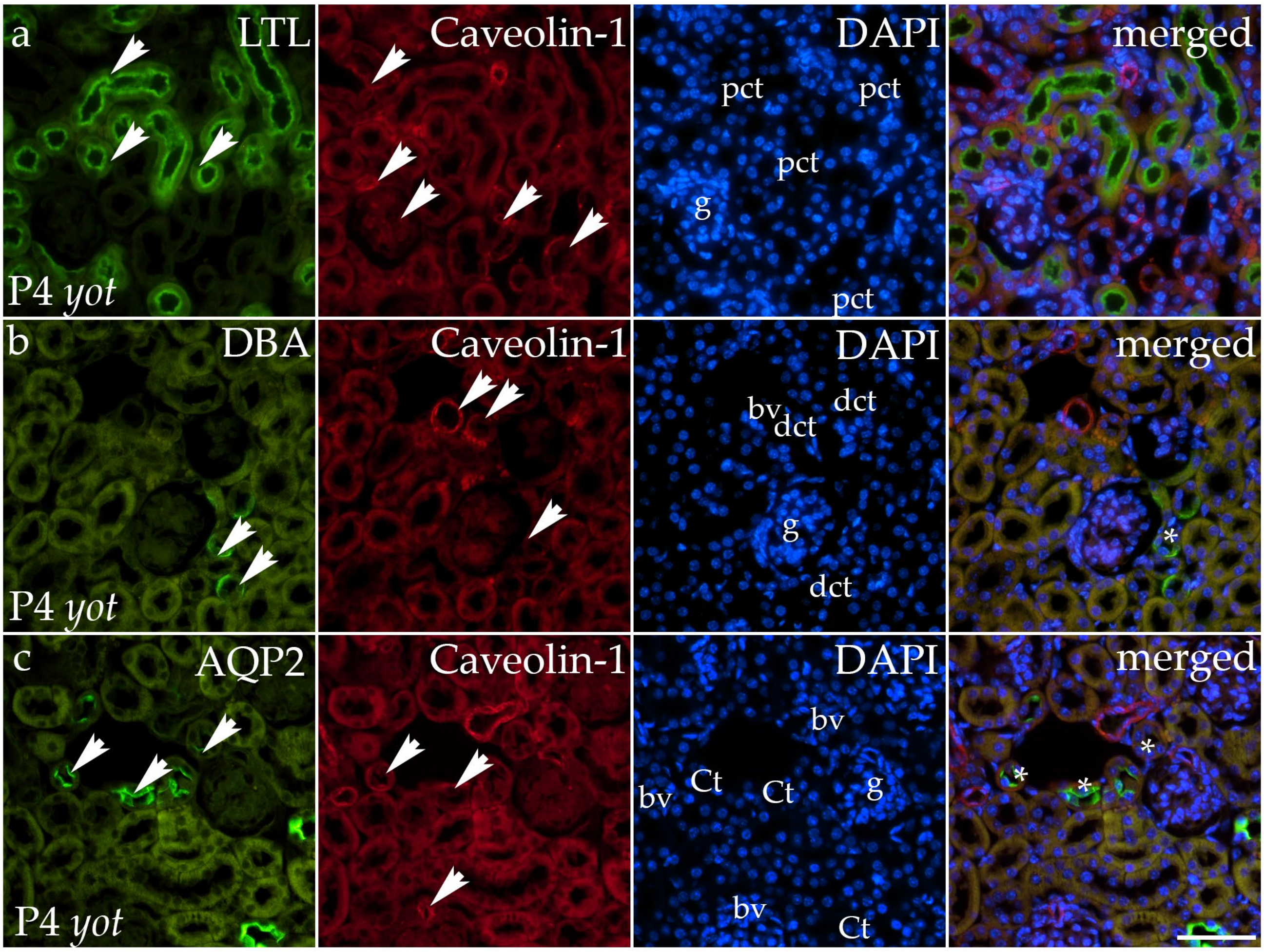

Figure S6. Double immunofluorescence staining of postnatal day 4 (P4) *yotari* (*yot*) mouse kidney cortex of the *Lotus tetragonolobus* lectin (LTL), a marker for proximal tubules (**a**), *Dolichos biflorus* agglutinin (DBA), a marker for distal tubules (**b**), and Aquaporin 2 (AQP2), a marker for distal and collecting tubules (**c**) with Caveolin-1 (**a**–**c**) marker. Arrows show the expression pattern of LTL, DBA, AQP2 and Caveolin-1 in glomeruli (g), proximal convoluted tubules (pct), distal convoluted tubules (dct), blood vessels (bv) and collecting tubules (Ct) indicated on 4′,6-diamidino-2-phenylindole (DAPI) nuclei staining image. An asterisk on merged LTL, DBA, AQP2, Caveolin-1 and DAPI image denotes the substructure where co-expression was detected. Images were taken on magnification ×40. The scale bar is 50 μm, which refers to all images.

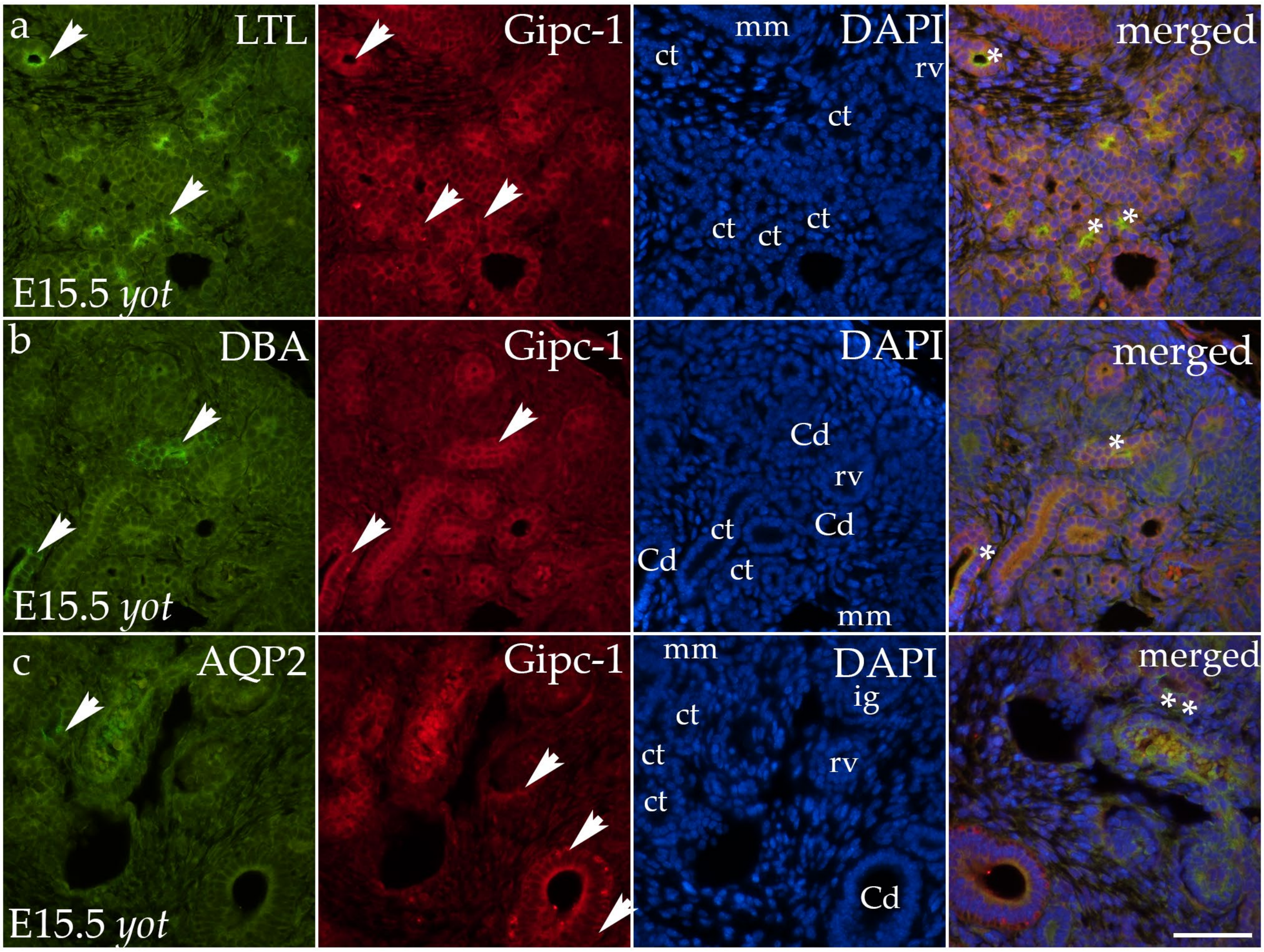

Figure S7. Double immunofluorescence staining of embryonic day 15.5 (E15.5) *yotari* (*yot*) mouse kidneys of the *Lotus tetragonolobus* lectin (LTL), a marker for proximal tubules (**a**), *Dolichos biflorus* agglutinin (DBA), a marker for developing renal collecting ducts (**b**), and Aquaporin 2 (AQP2), a marker for collecting ducts (**c**) with Gipc-1 (**a**–**c**) marker. Arrows show the expression pattern of LTL, DBA, AQP2 and Gipc-1 in metanephric mesenchyme (mm), renal vesicles (rv), glomeruli (g), convoluted tubules (ct), ampullae (A), and collecting ducts (Cd) indicated on 4′,6-diamidino-2-phenylindole (DAPI) nuclei staining image. An asterisk on merged LTL, DBA, AQP2, Gipc-1 and DAPI image denotes the substructure where co-expression was detected. Images were taken on magnification ×40. The scale bar is 50 μm, which refers to all images.

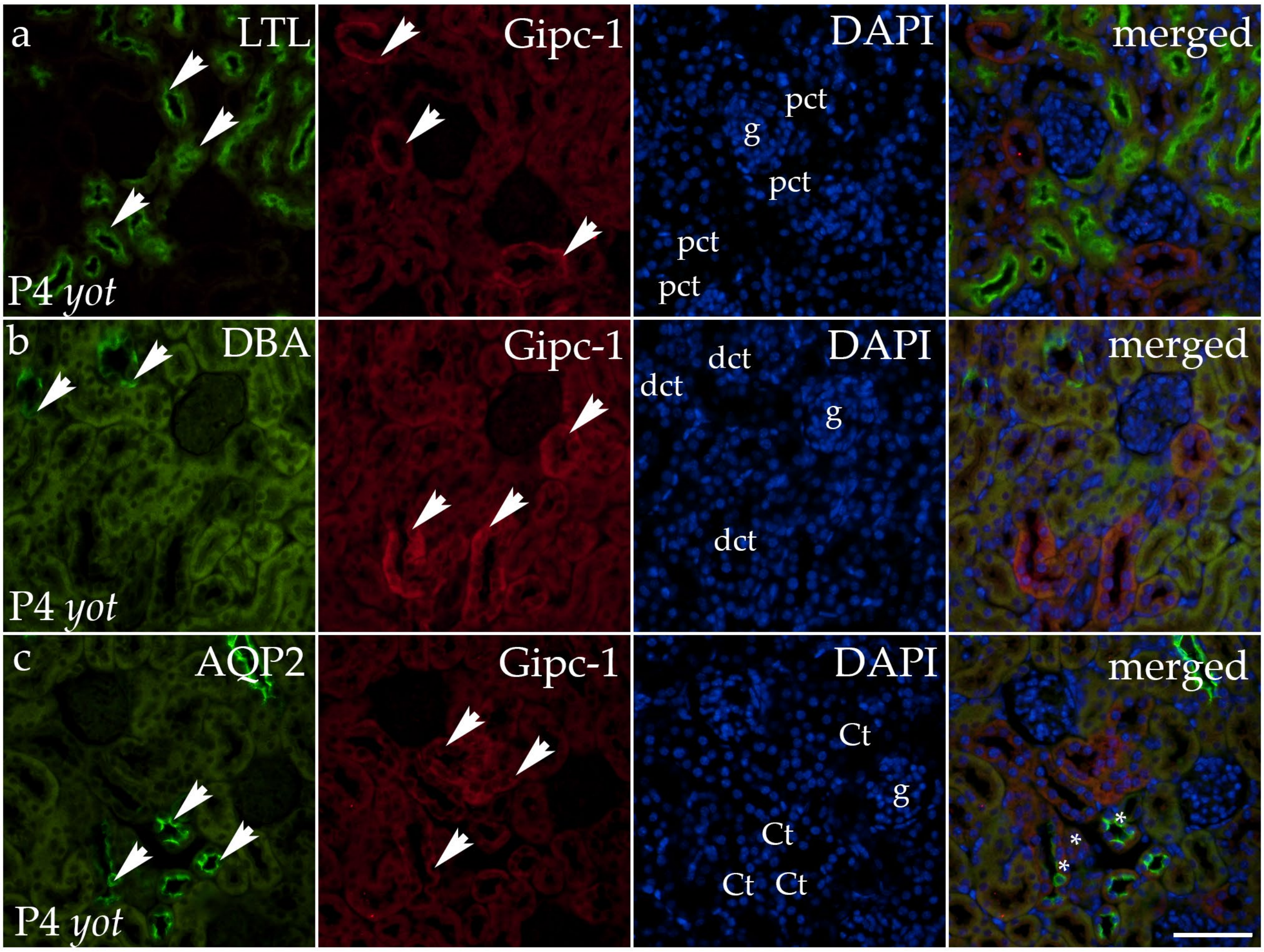

Figure S8. Double immunofluorescence staining of postnatal day 4 (P4) *yotari* (*yot*) mouse kidney cortex of the *Lotus tetragonolobus* lectin (LTL), a marker for proximal tubules (**a**), *Dolichos biflorus* agglutinin (DBA), a marker for distal tubules (**b**), and Aquaporin 2 (AQP2), a marker for distal and collecting tubules (**c**) with Gipc-1 (**a**–**c**) marker. Arrows show the expression pattern of LTL, DBA, AQP2 and Gipc-1 in glomeruli (g), proximal convoluted tubules (pct), distal convoluted tubules (dct) and collecting tubules (Ct) indicated on 4′,6-diamidino-2-phenylindole (DAPI) nuclei staining image. An asterisk on merged LTL, DBA, AQP2, Gipc-1 and DAPI image denotes the substructure where co-expression was detected. Images were taken on magnification ×40. The scale bar is 50 μm, which refers to all images.

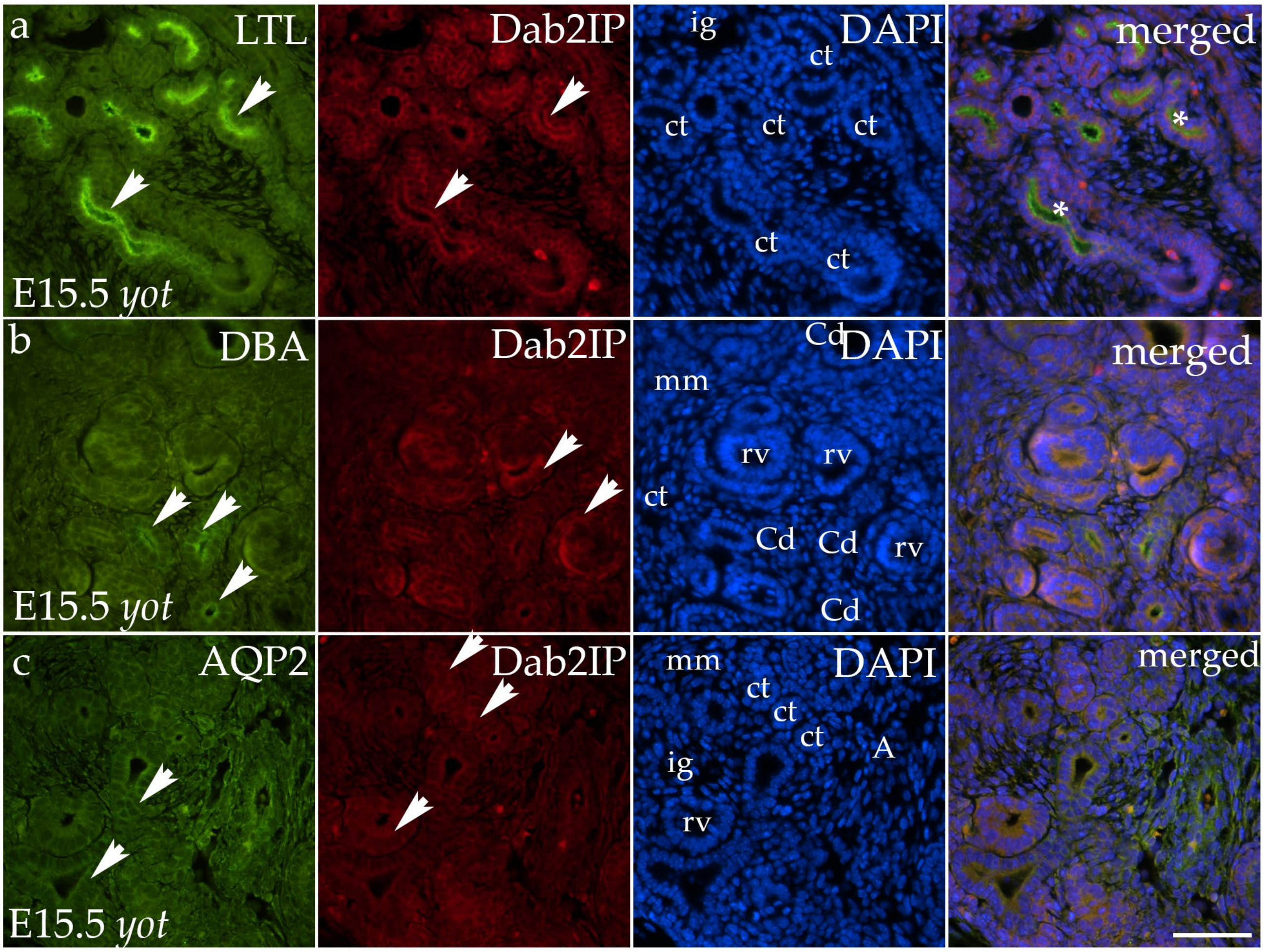

Figure S9. Double immunofluorescence staining of embryonic day 15.5 (E15.5) *yotari* (*yot*) mouse kidneys of the *Lotus tetragonolobus* lectin (LTL), a marker for proximal tubules (**a**), *Dolichos biflorus* agglutinin (DBA), a marker for developing renal collecting ducts (**b**), and Aquaporin 2 (AQP2), a marker for collecting ducts (**c**) with Dab2IP (**a**–**c**) marker. Arrows show the expression pattern of LTL, DBA, AQP2 and Dab2IP in metanephric mesenchyme (mm), renal vesicles (rv), glomeruli (g), convoluted tubules (ct), ampullae (A), and collecting ducts (Cd) indicated on 4′,6-diamidino-2-phenylindole (DAPI) nuclei staining image. An asterisk on merged LTL, DBA, AQP2, Dab2IP and DAPI image denotes the substructure where co-expression was detected. Images were taken on magnification ×40. The scale bar is 50 μm, which refers to all images.

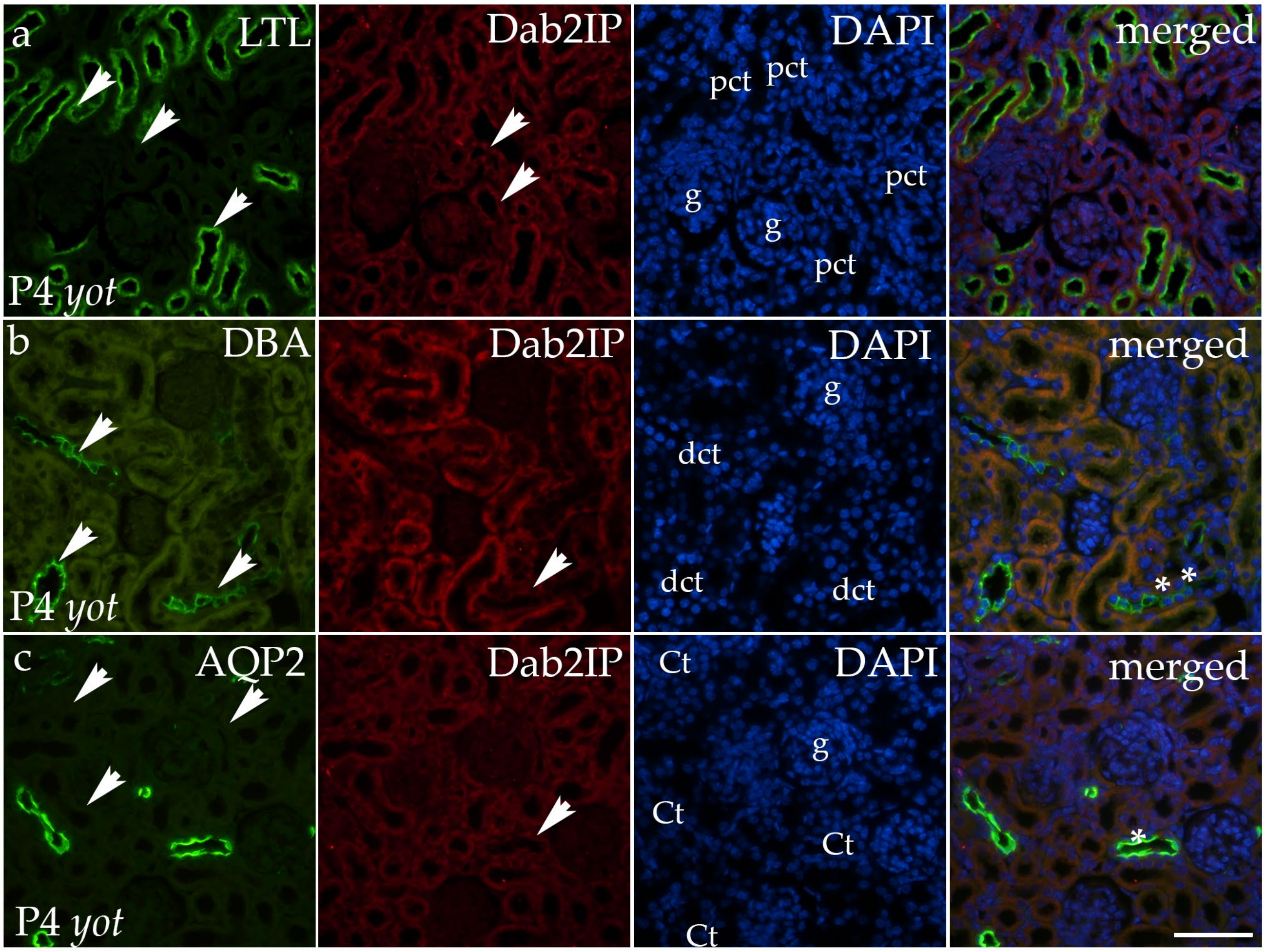

Figure S10. Double immunofluorescence staining of postnatal day 4 (P4) *yotari* (*yot*) mouse kidney cortex of the *Lotus tetragonolobus* lectin (LTL), a marker for proximal tubules (**a**), *Dolichos biflorus* agglutinin (DBA), a marker for distal tubules (**b**), and Aquaporin 2 (AQP2), a marker for distal and collecting tubules (**c**) with Dab2IP (**a**–**c**) marker. Arrows show the expression pattern of LTL, DBA, AQP2 and Dab2IP in glomeruli (g), proximal convoluted tubules (pct), distal convoluted tubules (dct) and collecting tubules (Ct) indicated on 4′,6-diamidino-2-phenylindole (DAPI) nuclei staining image. An asterisk on merged LTL, DBA, AQP2, Dab2IP and DAPI image denotes the substructure where co-expression was detected. Images were taken on magnification ×40. The scale bar is 50 μm, which refers to all images.

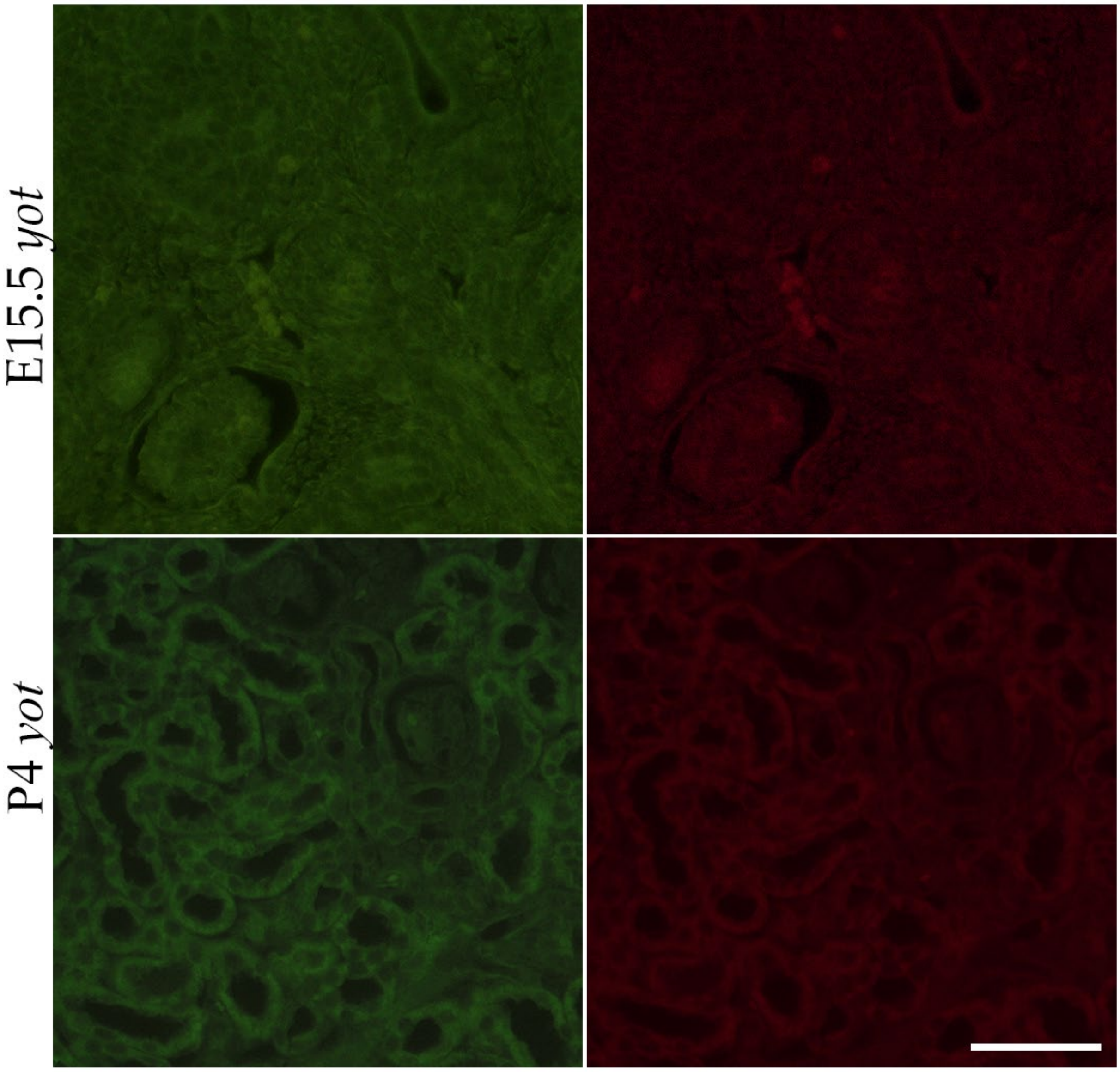

Figure S11. Negative control staining images (primary antibodies were omitted from the immunofluorescent protocol and only secondary antibody were applied on the sections) of embryonic day 15.5 (E15.5) *yotari* (*yot*) mouse kidneys and postnatal day 4 (P4) *yotari* (*yot*) mouse kidney cortex. Images were taken on magnification ×40. The scale bar is 50 μm, which refers to all images.
